# Obtaining and Characterization of Nutraceuticals Based on Linoleic Acid Derivatives Obtained by Green Synthesis and Their Valorization in the Food Industry

**DOI:** 10.3390/nu17152416

**Published:** 2025-07-24

**Authors:** Cristina Adriana Dehelean, Casiana Boru, Ioana Gabriela Macașoi, Ștefania-Irina Dumitrel, Cristina Trandafirescu, Alexa Ersilia

**Affiliations:** 1 Faculty of Food Engineering, Banat’s University of Agricultural Sciences and Veterinary Medicine “King Michael I of România”, 300645 Timisoara, Romania; cadehelean@umft.ro (C.A.D.); ersiliaalexa@usab-tm.ro (A.E.); 2 Research Center for Pharmaco-Toxicological Evaluations, “Victor Babes” University of Medicine and Pharmacy Timisoara, 300041 Timisoara, Romania; stefania-irina.dumitrel@umft.ro; 3 Faculty of Pharmacy, “Victor Babes” University of Medicine and Pharmacy Timisoara, 300041 Timisoara, Romania; trandafirescu.cristina@umft.ro; 4 Department of Medicine, Vasile Goldis Western University of Arad, Street Liviu Rebreanu, No. 86, 310048 Arad, Romania; boru.casiana@uvvg.ro

**Keywords:** nutraceuticals, linoleic acid, green synthesis, food industry

## Abstract

**Background/Objectives:** As an essential polyunsaturated fatty acid, linoleic acid (LA) plays an important role in maintaining the integrity of cellular membranes, modulating inflammatory responses, and mediating intracellular signaling. This review explores the structure, properties, and nutritional significance of LA and its bioactive derivatives, with particular attention to sustainable production methods and their potential applications. **Methods:** A comprehensive review of the recent literature was conducted, emphasizing the use of green synthesis techniques, such as enzyme-catalyzed biocatalysis and microbiological transformations, in order to obtain LA-derived nutraceuticals. Analyses were conducted on the key aspects related to food industry applications, regulatory frameworks, and emerging market trends. **Results:** Through green synthesis strategies, LA derivatives with antioxidant, anti-inflammatory, and antimicrobial properties have been developed. There is potential for these compounds to be incorporated into health-oriented food products. In spite of this, challenges remain regarding their stability and bioavailability. Furthermore, there are inconsistencies in international regulatory standards which prevent these compounds from being widely adopted. **Conclusions:** The development of functional and sustainable food products based on linoleic acid derivatives obtained using ecological methods offers significant potential. Research is required to optimize production processes, enhance compound stability, and clinically validate health effects. The integration of the market and the safety of consumers will be supported by addressing regulatory harmonization.

## 1. Introduction

The term “nutraceuticals” was first mentioned by Dr. Stephen DeFelice in 1989, and it combines the words “nutrition” and “pharmaceutical.” The word “nutraceuticals” is a syncretic neologism that refers to the fact that various products obtained from different sources (herbal products, dietary supplements, processed foods, beverages) are used as medicine rather than for their nutritional role. In a nutshell, a nutraceutical is a product isolated from food that is believed to help in the prophylactic and curative treatment of diseases [1,2].

A healthy diet is a major component in extending the health span and preventing non-communicable diseases (NCDs), such as cancer, heart-related problems, diabetes, obesity, neurological and mental health disorders, as well as age-related diseases [3]. The World Health Organization (WHO) estimated that approximately 70% of the total 57 million deaths in 2016 were due to NCDs [4]. The WHO’s approach to preventing and controlling NCDs includes strategies for addressing unhealthy diet patterns and lowering behavioral risk factors (compulsive eating, sedentarism, nicotine consumption, excessive alcohol use). The WHO recommends a balanced diet consisting of a limited consumption of saturated and trans fats, with a higher intake directed toward unsaturated fats, a diet that includes more fruits and vegetables, and a limited intake of salt and sugar [3,5]. Additionally, a healthy diet consists not only of what food is consumed, but also of how, why, and when the food is consumed. Due to the challenging times we live in, life can often be stressful, and because of that, several behavioral risks can be attributed to an unhealthy diet, such as emotional eating, eating too fast, having a restrictive diet, and the amount of food consumed [5]. Because it can be quite hard for the majority of people to find a nutritious diet, sometimes nutraceuticals are a life-changing alternative. Although nutraceuticals can help to fill in the gaps in an unbalanced diet, they must be taken with additional nourishment, as they are not sufficient to complete the daily intake of nutrients [2]. Nutraceuticals have gained outstanding popularity in recent years due to their ability to improve overall health, prevent the onset of diseases, delay aging, prolong the lifespan, and support body function. The global nutraceutical market is predicted to grow by 9% from 2021 to 2030, and it is estimated to reach almost USD 405 billion by 2025 [6]. One of the key mechanisms through which nutraceuticals exert therapeutic effects in various diseases (e.g., cardiovascular diseases; gastrointestinal, renal, and neurological disorders; diabetes; atherosclerosis; cancer) is due to their ability to counteract the reactive oxygen species, reduce inflammation, and balance the redox state [7,8].

In these difficult times, when the prevalence of chronic diseases is on the rise, diet is a crucial factor that mediates the occurrence and development of multiple diseases. Polyunsaturated fatty acids (PUFAs) are among the most important dietary components, playing a crucial role in the structure and function of cellular membranes. As essential fatty acids, PUFAs are involved in various biological processes, including cell signaling, gene expression regulation, and brain function. They also serve as endogenous mediators that contribute to membrane integrity and optimal cellular activity. Among the essential polyunsaturated fatty acids of the omega-6 class, linoleic acid (LA) is an essential component of maintaining human health. It contributes to a number of physiological processes, including maintaining the integrity of cell membranes, regulating inflammation, and maintaining cardiovascular function. The remarkable bioactive properties of this compound have recently attracted increasing interest from the scientific community [9,10].

The integration of sustainable synthesis methods is a crucial aspect of the production of nutraceuticals. As nutraceuticals aim to improve health and prevent chronic diseases, the process of creating these health-promoting compounds must align with principles that ensure environmental, social, and economic sustainability [11].

This review aims to provide an overview of LA derivatives obtained by green synthesis, highlighting their potential for valorization in the food industry as health-promoting ingredients, along with considerations of their safety profile.

## 2. Linoleic Acid: Structure, Properties, and Biological Significance

### 2.1. Chemical Structure and Physicochemical Properties

**Figure 1:**
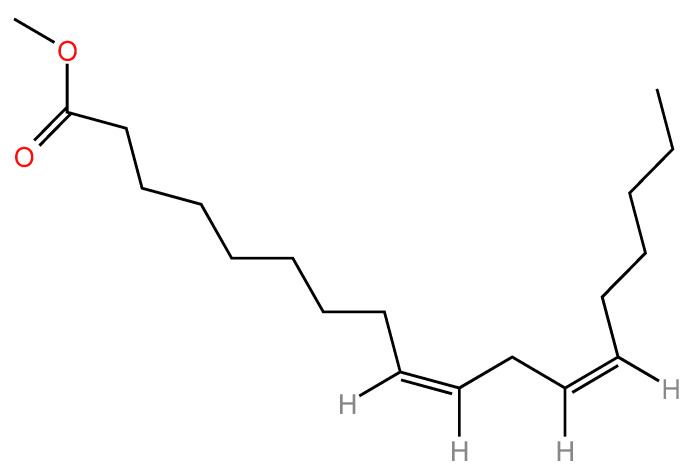
Linoleic acid chemical structure. The molecular structure was made with KingDraw, V3.6.1.

Linoleic acid (Figure 1) is the parent of the family of omega-6 fatty acids and is the most abundant source of polyunsaturated fatty acids (PUFAs) [12].

It plays an essential role in maintaining the structural and functional integrity of cell membranes. Chemically, LA possesses the molecular formula C_18_H_32_O_2_, with a molecular mass of 280.45 g per mole. The structure consists of an aliphatic chain with 18 carbon atoms, which contains two double bonds of cis-configuration at positions Δ9 and Δ12 (cis-9, cis-12-octadecadienoic acid). As a result of this particular arrangement, LA is in a zigzag arrangement with large torsion angles, which significantly affects the rigidity of the carbon chain and reduces its melting point to −5 degrees Celsius, making it liquid at room temperature [13,14].

With a hydrophobic hydrocarbon region and a polar carboxyl end, linoleic acid exhibits physicochemical properties indicative of its amphipathic nature [15]. Due to the predominance of the non-polar region, LA is practically insoluble in water, but is very soluble in non-polar and weakly polar organic solvents such as hexane, chloroform, diethyl ether, and ethanol. Consequently, it can be used in a wide range of industrial and pharmaceutical applications due to its solubility [16,17].

Two double bonds conjugated by a bis-allylic hydrogen system (located between the two double bonds) provide the molecule with a high degree of oxidative susceptibility, especially when oxygen, light, and transition metals are present [18]. Consequently, lipid peroxidation results in an impairment of the oxidative stability of LA and the formation of unstable secondary compounds (aldehydes, ketones), which adversely affect the quality of the food and pharmaceutical products in which it is used. Thus, stabilizing LA by microencapsulation, adding antioxidants, or making structural modifications is an active research area [13,14,19,20,21].

### 2.2. Biological Functions and Health Benefits

Linoleic acid plays a critical role in the structure, metabolism, and signaling functions of cells. Due to its status as an essential fatty acid, it cannot be synthesized by the body and must be obtained through diet alone [10,22].

Molecularly, linoleic acid constitutes an essential component of membrane phospholipids, especially phosphatidylcholine and phosphatidylethanolamine. In the sn-2 position of phospholipids, it influences such aspects as fluidity and permeability, as well as the organization of microdomains, such as lipid bubbles, which affect the biophysical properties of the membrane. In addition to their role in protein sorting, signal transduction, and endocytosis, these microdomains play an essential role in cell communication and the maintenance of cell homeostasis [13,23].

Through the process of desaturation and elongation, LA acts as a biochemical precursor in the biosynthesis of long-chain polyunsaturated fatty acids (PUFAs). LA is converted to gamma-linoleic acid (GLA) by the enzyme 6-desaturase, which subsequently undergoes elongation and desaturation to obtain dihomo-gamma-linoleic acid (DGLA) and arachidonic acid (AA). These derivatives are used as substrates by the enzymes cyclooxygenase (COX), lipoxygenase (LOX), and cytochrome P450, which facilitate the production of eicosanoids that are responsible for controlling inflammation, vasodilation, platelet aggregation, and immune responses, such as prostaglandins, leukotrienes, and thromboxanes [24,25].

The anti-inflammatory effects of LA are mainly attributed to its conversion to DGLA, which is further converted to PGE_1_, known to have anti-inflammatory and immunoregulatory activity [26,27,28]. LA acts as a precursor to arachidonic acid (AA), which is subsequently transformed into eicosanoids, which play key roles in inflammation, immune responses, and various physiological functions. These eicosanoids include prostaglandins (PGE_2_), thromboxanes (TXA_2_), and leukotrienes (LTB_4_). Although these are known to be proinflammatory mediators, some eicosanoids present a dual nature. For example, PGE_2_ can induce an inflammatory response by promoting leukotriene production through the inhibition of 15-lipoxygenase. Conversely, it can also elicit an anti-inflammatory response by stimulating the activity of 15-lipoxygenase [29].

As a crucial component of maintaining the skin’s barrier function, LA is also essential for the synthesis of ceramides, which are key lipids in the stratum corneum and have the ability to repair the skin barrier, as well as prevent water loss and protect the skin from environmental exposure. Dysfunctions in LA metabolism are linked to the development of various skin conditions and diseases, such as psoriasis, atopic dermatitis, acne, and cancer [30,31,32].

When saturated fats in the diet are replaced with LA, there may be positive effects on cardiovascular health, such as lowering LDL cholesterol and potentially lowering heart disease risk [33]. A large meta-analysis of 13 prospective cohort studies, including a total of 310,602 individuals, examined the relationship between LA and coronary heart disease (CHD) risk. Among these participants, 12,479 CHD events and 5882 CHD-related deaths were recorded. The findings indicated that a 5% increase in energy intake from LA was linked to a 10% reduction in the risk of CHD events and a 13% decrease in CHD-related mortality [34].

As a potential treatment for type 2 diabetes and metabolic syndrome, LA has been widely acknowledged. Research suggests that higher dietary intake of LA-rich foods is linked to improved insulin sensitivity, improved glucose metabolism, reduced ectopic fat accumulation (particularly in visceral adipose tissue and liver), and lower inflammation, all of which contribute to the prevention and management of type 2 diabetes and metabolic syndrome [35,36,37,38,39,40,41,42].

Additionally, during critical growth phases, such as infancy, LA and other essential fatty acids are necessary for neural development and overall health [33,43]. However, excessive intake of omega-6 fatty acids relative to omega-3s has been associated with inflammatory conditions, sparking ongoing debates about the ideal dietary balance [29].

### 2.3. Dietary Sources and Importance in Human Nutrition

LA is widely distributed in various dietary sources, including vegetable oils, nuts, seeds, and animal products. In Table 1, some of the food sources of LA, categorized by food type, and their approximate LA content per 100 g are presented [9,10,44,45,46,47,48,49].

LA plays a vital role in human nutrition because it cannot be synthesized by the body and must be obtained through dietary sources. It is the most commonly consumed PUFA, predominantly consumed in modern Western diets, serving both as a source of energy and a key structural component of cell membranes. To support optimal body function and development, it is recommended that LA comprises 1% to 2% of daily energy intake, although currently, LA accounts for >7% of daily calories. LA is crucial for the structural integrity and fluidity of cell membranes. It is part of membrane phospholipids and contributes to membrane permeability and fluidity, which are essential for proper cellular function and communication [43]. Due to LA’s role as a signaling mediator and gene expression regulator, it is directly involved in maintaining brain function [12]. LA is a precursor of oxidized linoleic acid metabolites (OXLAMs), which are abundant in peripheral tissue and are known lipid mediators that regulate pain and inflammation [43]. Additionally, by its conversion to GLA, it serves as a structural component of neuronal membrane phospholipids, important for neural blood flow [50]. While LA is important in human nutrition, as it provides a number of benefits, excessive intake without adequate omega-3 PUFAs can alter inflammatory pathways and increase the development of some diseases [29]. For example, it has been suggested that higher maternal LA levels during pregnancy may be associated with neurodevelopmental challenges in children, having a greater risk of developing autistic traits by age 6, and doubling the risk of delayed psychomotor and mental development in 6-month-old infants. Therefore, maintaining a balanced intake of LA is critical to maximize its benefits while minimizing potential harm [51,52].

## 3. Green Synthesis of Linoleic Acid Derivatives

### 3.1. Principles of Green Chemistry in Synthesis

The concept of green chemistry in synthesis was developed by Paul Anastas and John Warner in the early 1990s. In recent years, this field has drawn the attention of researchers as an alternative to conventional synthetic methods, with the major advantage of reducing waste, energy consumption, and the use of potentially toxic chemicals. As a conceptual foundation for sustainable chemical synthesis, green chemistry is guided by twelve guiding principles [53].

Among the most important principles of green synthesis is the prevention of waste. This principle has significant implications for many areas, including agri-food, where excessive agrochemical residues can result in contamination of water and soil [54]. A second concept related to green synthesis is atom economy. Through the optimization of synthetic pathways, all reactants are incorporated into the final product, thereby improving resource efficiency [55]. Specifically, this principle contributes to minimizing resource depletion, improving yield purity, and eliminating some of the steps associated with purification in nutraceutical manufacturing [56]. An important aspect of green chemistry involves the application of milder reaction conditions and biotechnological approaches in order to reduce the occurrence of toxic intermediates and contribute to the development of safer nutraceuticals for human consumption [57]. Furthermore, this principle contributes to the production of biodegradable and effective products that are free of harmful substances. This area is particularly relevant to the use of safer solvents with regard to their toxicological profiles [58]. There are several techniques that are used in the process, such as water extraction and supercritical CO_2_, that are safer alternatives to conventional solvents, which consequently improves the purity of the final product by reducing the residue on the final product [59,60]. Moreover, enzymatic catalysis and photochemical synthesis reduce energy consumption while maintaining the product’s efficacy [61,62]. One of the hottest topics in green chemistry is the use of renewable raw materials. As an example, agricultural waste can be used as a raw material for producing nutraceuticals, reducing dependence on finite resources and improving circular economy models [63,64]. Nutraceuticals are produced using the green synthesis method by applying simplified extraction and purification procedures in order to retain the natural bioactivity of plant-derived compounds [65]. Among the most effective methods is the use of catalysis, which has the advantage of improving the specificity of the reaction and reducing the waste of reagents and unwanted byproducts. In the production of nutraceuticals, biocatalysis enables regioselective transformations in order to preserve the bioactivity of the compounds [66]. Another basic principle in green chemistry is that compounds should be metabolized and eliminated efficiently from the human body as well as the environment, thereby ensuring a minimum amount of pollution. The application of real-time analysis for pollution prevention enhances quality control in this regard [67,68]. Spectroscopic and chromatographic techniques allow continuous monitoring of synthesis processes, ensuring that unwanted byproducts are minimized and product purity is maintained [69,70]. A final but equally important aspect involves the replacement of potentially flammable or highly reactive substances with safer alternatives, thereby reducing the risks associated with product handling and improving the product’s safety profile [71].

In order to meet the growing demand for sustainable and environmentally friendly processes in the field, green synthesis principles are being applied in the production of nutraceuticals derived from linoleic acid derivatives. Several effective and selective pathways exist for the transformation of linoleic acid into bioactive derivatives with improved functional properties, including enzyme-catalyzed reactions, microbial biotransformations, and solvent-free or low-toxicity solvent-based chemical modifications [72,73]. By using these approaches, toxic wastes are reduced, energy consumption is reduced, and finished products are purer, making them suitable for nutraceutical applications. The following sections provide an overview of specific green synthesis methods for linoleic acid derivatives, with an emphasis on their potential to be used to develop high-quality nutraceuticals with health-promoting properties [74].

### 3.2. Eco-Friendly Catalysts, Solvents, and Processes

The conventional method of synthesis often utilizes toxic reagents, non-renewable resources, and energy-intensive conditions, which pose risks both in terms of human health and environmental pollution. In contrast, green synthesis emphasizes the use of biodegradable solvents, non-toxic catalysts, and energy-efficient reactions [75,76].

The use of green catalysts, such as biocatalysts, enzymes, and organocatalysts, enables selective transformations with high efficiency [77,78]. Moreover, the use of green solvents, such as water, supercritical fluids, ionic liquids, or deep eutectic solvents, reduces pollution and is a safer and more sustainable alternative to volatile organic compounds [79,80]. The development of sustainable chemistry processes, such as microwave or ultrasound-assisted synthesis and flow chemistry, contributes significantly to the improvement of reaction yields and the reduction of energy consumption [81].

Green chemistry contributes to safer pharmaceuticals, nutraceuticals, and biomaterials with an increased focus on both economic viability and environmental responsibility through the use of green solvents, catalysts, and processes [82,83].

Current trends have focused on achieving and implementing sustainable and environmentally friendly chemical processes, resulting in significant advancements in green synthesis, particularly in the nutraceutical industry. In the development of nutraceuticals, linoleic acid and its derivatives have attracted attention due to their biological properties [84,85]. Using green synthesis principles to produce linoleic acid derivatives, including green catalysts, green solvents, and energy-efficient processes, offers an alternative method for obtaining compounds with high biological value while minimizing environmental impact [84].

#### 3.2.1. Eco-Friendly Catalysts in Green Synthesis of Linoleic Acid Derivatives

In order to obtain linoleic acid derivatives, catalysts are crucial, as they are involved in selective and efficient reactions under mild conditions [86]. The most widely used eco-friendly catalysts in the nutraceutical field are discussed below.

##### Enzyme-Based Catalysts (Biocatalysts)

There are numerous types of biocatalysts available, such as lipases, lipoxygenases, and microbial enzymes, which are involved in the specificity and efficiency of reactions, while functioning under mild, ambient conditions [87,88,89].

Lipases include lipases from *Candida antarctica* and *Rhizomucor miehei* [90]. These enzymes are widely used to transform linoleic acid into structured lipids with increased bioavailability by esterifying it with glycerol, ethanol, or other polyols enzymatically. A major advantage of these reactions is that they occur in an aqueous or solvent-free environment, which eliminates the need for dangerous acid catalysts [91].

Various lipoxygenases, including those found in soybeans or microorganisms, catalyze the regioselective oxidation of linoleic acid to hydroperoxides, producing hydroxy and keto derivatives, including 9-hydroxy and 13-hydroxy-octadecadienoic acids (HODE), which have antioxidant and anti-inflammatory effects [92,93].

Certain strains of *Lactobacillus* and *Bifidobacterium* produce microbial isomerases that play a role in the transformation of linoleic acid into conjugated linoleic acid isomers, which have been implicated in lipid metabolism and immunomodulation [94,95].

##### Heterogeneous Catalysts

The advantages of heterogeneous catalysts are high recyclability, stability, and low environmental toxicity compared to conventional acid and base catalysts [96,97].

Titanium and zirconium oxides are involved in the epoxidation of linoleic acid, utilizing hydrogen peroxide as an oxidant and replacing the hazardous conventional peracid-based method. During this reaction, epoxides are generated, which are further used as precursors to produce biodegradable polymers and formulations [98].

It has been shown that montmorillonite clay-based catalysts, such as natural and modified montmorillonite clays, act as solid acid catalysts in the hydroxylation and oxidation reactions of linoleic acid under mild conditions, leading to the production of bioactive lipid derivatives [99,100].

In nutraceutical applications, gold and palladium nanoparticles are capable of catalyzing the hydrogenation of linoleic acid, ensuring oxidative stability while retaining functional properties [101,102].

##### Organocatalysts

Organocatalysts are used in green reactions to catalyze the reaction, and they offer the advantage of conducting the transformation without the use of toxic metals [103].

The esterification of linoleic acid with functional polyols can be carried out with ionic liquids such as deep eutectic solvents based on choline chloride. Such ionic liquids serve as both catalysts and reaction media, yielding high-purity nutraceutical esters from linoleic acid [104,105].

In linoleic acid intermediates, proline catalysts promote the formation of carbon–carbon bonds, leading to the formation of bioactive compounds with high antioxidant properties [106].

#### 3.2.2. Green Solvents in Linoleic Acid Derivative Synthesis

A basic principle of green synthesis is the use of environmentally friendly solvents to replace volatile organic compounds in chemical processes [57].

The use of supercritical CO_2_ as a tunable solvent has implications for the enzymatic and catalytic transformation of linoleic acid, as it facilitates esterification and oxidation reactions under mild conditions with easy solvent recovery [107].

It has been demonstrated that water-based systems, such as aqueous enzymatic reactions, offer the advantages of minimal environmental impact and improved selectivity in lipid modification, especially esterification and hydrolysis [108,109].

As an alternative to toxic organic solvents in esterification and transesterification reactions, deep eutectic solvents are used, such as choline chloride compounds or biodegradable hydrogen bond donors, which have the advantage of high catalytic efficiency and improved biocompatibility [110].

#### 3.2.3. Sustainable Processes in Linoleic Acid Derivative Synthesis

Several breakthroughs in the field of green chemistry have allowed new sustainable reaction methods to be developed that reduce energy consumption and the generation of toxic waste [111].

In microwave-assisted synthesis, such as microwave irradiation, enzymatic and catalytic reactions are accelerated and molecular interactions are improved, significantly reducing the reaction times for linoleic acid derivatization [112,113].

By applying ultrasound-assisted catalysis under solvent-free conditions, emulsification and mass transfer can be promoted, while esterification and enzymatic oxidation reactions can be increased in efficiency [114].

Flow chemistry involves the use of continuous flow reactors, which are used to control the rate at which linoleic acid derivatives are synthesized. These reactors reduce solvent waste while improving reaction yields and enabling controlled and efficient synthesis of linoleic acid derivatives [115].

### 3.3. Enzymatic vs. Chemical Synthesis Approaches

In the synthesis of compounds with biological activity, including nutraceuticals and pharmaceuticals, as well as biomaterials for medical applications, a wide range of methodologies has been employed. This includes conventional chemical syntheses as well as green syntheses, such as enzymatic syntheses [116,117]. Although both approaches result in compounds with applications in various fields, their selectivity, environmental impact, reaction conditions, and process efficiency differ significantly [118].

Traditionally, chemical synthesis has dominated the production of bioactive compounds, utilizing synthetic reagents, catalysts, and harsh reaction conditions involving high temperatures or the use of acids, bases, and toxic organic solvents [119]. Chemical synthesis has the advantage of high reaction speed and wide applicability. However, it lacks substrate specificity and generates undesirable byproducts that can pollute the environment and have negative health impacts [120].

As an alternative, enzymatic syntheses are carried out with enzymes such as lipases, hydrolases, and oxidoreductases that provide selective transformations under mild reaction conditions [77]. There are several advantages of these approaches when it comes to the synthesis of nutraceuticals, including high substrate specificity, low energy requirements, and improved atom economy, making them an appealing alternative for sustainable and environmentally friendly chemical applications [121]. Further, biocatalysts can operate in an aqueous or an alternative green solvent system, reducing the need for volatile organic compounds and the amount of toxic waste produced [122]. Despite the advantages of this approach, it has several disadvantages, including problems associated with enzyme stability, increased costs, and scalability challenges [123].

#### 3.3.1. Chemical Synthesis of Linoleic Acid Derivatives

Synthesis of linoleic acid derivatives involves acid- or base-catalyzed transformations, oxidations or hydrogenations, and thermal isomerization [124]. These methods have the advantage of high reaction rates and scalability, which makes them perfect for industrial applications [120]. There are, however, undesirable byproducts and environmental damage from toxic reagents and harsh reaction conditions [125].

##### Conjugated Linoleic Acid (CLA) Production

There is a number of methods available for obtaining CLA, including the use of strong bases such as potassium hydroxide or sodium hydroxide. The reactions take place under harsh conditions, such as high temperatures above 180 °C, in organic solvents, with high conversion rates but low isomer selectivity [113,126]. Alternatively, transition metal catalysts such as Ru, Pt, and Pd can facilitate hydrogenation and selective double bond migration. As a result of this method, a greater degree of selectivity can be achieved; however, the use of metal catalysts often results in toxic residues that must be removed through subsequent purification steps [127].

##### Epoxidation of Linoleic Acid

The epoxidation of linoleic acid may be performed on a peracid basis by using m-chloroperbenzoic acid or performic acid. Both acids oxidize the double bonds of linoleic acid and lead to the formation of epoxides, which can be used to prepare hydroxylated derivatives. In addition to the advantage of being very efficient, this reaction also requires the use of corrosive oxidizers and extensive downstream processing in order to eliminate the peracid residues [128,129]. As an alternative, TiO_2_- or ZrO_2_-supported catalysts can be used in the presence of H_2_O_2_ for catalytic epoxidation, which is a more environmentally friendly method with a lower environmental impact [130].

##### Esterification and Transesterification

In acid-catalyzed esterification, strong acids such as sulfuric acid are used to catalyze the esterification reaction between linoleic acid and glycerol, ethanol, or sterols to produce functional lipids. In spite of the fact that these reactions are capable of producing high yields, they require high temperatures and the use of excess reagents, which generate acid waste streams [131].

The transesterification of linoleic acid involves the use of alkaline catalysts, such as NaOCH_3_ or KOH, facilitating the conversion of methyl or ethyl esters of linoleic acid into structured lipids [132].

#### 3.3.2. Enzymatic Synthesis of Linoleic Acid Derivatives

Enzymatic synthesis relies on the use of biocatalysts such as lipases, oxidoreductases, and microbial enzymes to achieve highly selective transformations under mild conditions [121]. The main advantages of these methods are increased regio- and stereoselectivity, low byproduct formation, and increased durability. Thus, these methods are highly suitable for the production of nutraceuticals [133].

##### Bioconversion to Conjugated Linoleic Acid (CLA)

In the production of CLA isomers, strains of *Lactobacillus*, *Bifidobacterium*, and *Propionibacterium* are used, which are involved in the transformation of linoleic acid by enzymes called bacterial reductases. The method has several advantages, including the fact that it is conducted under physiological conditions and produces a high level of purity without the use of harsh chemicals [94,134].

##### Enzymatic Epoxidation and Hydroxylation

Soybean lipoxygenases catalyze the oxidation of linoleic acid to 9- and 13-hydroxy derivatives. The advantage of this method is that it is carried out in water, which prevents lipid peroxidation. Furthermore, the specific epoxidation of linoleic acid is catalyzed by non-specific peroxygenases that act under mild conditions, thus avoiding toxic effects [135,136].

##### Lipase-Catalyzed Esterification and Transesterification

Under solvent-free conditions, *Candida antarctica* lipase B and *Rhizomucor miehei* lipase can catalyze the esterification of linoleic acid with ethanol, glycerol, or sterols to form bioactive esters. Transesterification by enzymes is catalyzed by lipase, which allows mild, regioselective modification of linoleic acid with increased reaction efficiency and reduced byproduct formation [137,138].

#### 3.3.3. Comparative Analysis and Industrial Implications

Some differences between chemical and enzymatic synthesis approaches are presented in Table 2.

### 3.4. Recent Advancements and Innovations in Green Synthesis

Based on the disadvantages associated with conventional chemical synthesis, green synthesis is emerging as a viable alternative in sustainable chemistry, with the goal of minimizing environmental and economic impacts. Recent advances in this field have been made possible by the use of renewable raw materials, biomimetic catalysts, energy-efficient reaction conditions, and the incorporation of nanotechnology [139].

#### 3.4.1. Catalytic Innovations in Green Synthesis

Biocatalysis is one of the subfields of green synthesis that has made significant progress in recent years. There is a continuous expansion of this field, in particular through the development of enzymatic engineering and the application of immobilization strategies [140]. Recent advances include genetically modifying enzymes involved in green synthesis processes, such as lipases and oxidases, in order to improve their stability, substrate specificity, and reusability in aqueous and non-aqueous media [141,142]. An important breakthrough was the development of artificial metalloenzymes, which have been numerically engineered from proteins and have enabled highly selective transformations to take place under mild conditions. In addition, recent studies have indicated that the integration of microfluidic reactors with enzymatic catalysis facilitates continuous-flow synthesis with reduced solvent consumption and improved catalytic efficiency [143,144].

As heterogeneous catalysts, metal–organic frameworks (MOFs) have captured the attention of researchers due to their advantages, such as tunable porosity, uniform surfaces, and modular functionality. Functionalized MOFs utilize abundant earth metals, such as iron or copper, to fix CO_2_ or to undergo hydrogenation or oxidative coupling reactions, thus eliminating the need for heavy metals [145,146]. A noteworthy advancement in this area is the incorporation of plasmonic nanoparticles into MOF arrays for visible light-driven catalysis, which significantly reduces the input of energy in synthetic processes [147,148].

#### 3.4.2. Solvent-Free and Alternative Solvent Systems

Green synthesis has been revolutionized by the development of green solvents, such as deep eutectic solvents (DES) and ionic liquids (ILs) [149,150]. There are several factors that contribute to the tunable physicochemical properties of DES, including their composition of naturally derived hydrogen bond donors and acceptors, and their negligible volatility. Among their main applications are biomass valorization, metal recovery, and nanoparticle synthesis [151,152,153]. ILs functionalized with catalytic fragments have also demonstrated improved recyclability and selectivity in multicomponent reactions [154,155,156].

Another approach to green synthesis, mechanochemical synthesis, for instance, via ball milling or ultrasound activation, can result in solvent-free and minimally energy-consuming transformations [157,158,159]. Through the use of in situ spectroscopy techniques, recent advances in the mechanistic understanding of reaction pathways have facilitated the rational design of solvent-free techniques for C–C and C–N bond-forming reactions and sustainable polymerization processes. A remarkable advancement has been made in the elimination of hazardous reactants by combining mechanochemistry with electrochemical methodologies [160,161].

#### 3.4.3. Sustainable Nanotechnology and Green Nanomaterials

In recent years, increased attention has been paid to the biosynthesis of metallic and bimetallic nanoparticles using plant extracts, microbial systems, and biomolecules as an alternative to conventional methods of synthesis [162,163,164]. The use of phytochemicals in the synthesis of nanoparticles has recently led to the production of size- and shape-controlled nanoparticles with improved catalytic, antimicrobial, and electronic properties. Functionalization of nanomaterials with biodegradable ligands has improved their stability and biocompatibility, extending their application in drug delivery and reducing environmental impact [165,166].

A growing interest in green synthesis has been shown in graphene derivatives, carbon nanotubes, and carbon dots used as catalysts. As a result of recent developments in microwave-assisted synthesis, defect-engineered carbon nanostructures have been produced with superior electron transport properties [167,168]. Additionally, biochar-derived nanomaterials synthesized from agricultural waste have demonstrated promising applications in photocatalysis and heavy metal adsorption, thus closing the loop in the circular economy paradigm [169,170].

#### 3.4.4. Energy-Efficient Strategies in Green Synthesis

In order to reduce the energy consumption of conventional synthetic processes, semiconductor photocatalysts and electrocatalytic systems are integrated. The absorption of visible light by photocatalysts based on TiO_2_ and perovskite derivatives has been optimized, allowing for photoredox catalysis in aqueous media [171]. Meanwhile, electrocatalytic hydrogenation and oxidative coupling have emerged as viable alternatives to classical reductive and oxidative transformations, eliminating the requirement for hazardous reagents and extreme reaction conditions [172,173].

Microwave irradiation is another method for improving reaction kinetics, mass transfer, and product yield in green synthesis. By combining these techniques with catalytic systems, it has been possible to synthesize heterocycles, biopolymers, and pharmaceutical intermediates under mild conditions [174,175]. There have been recent studies demonstrating scalable and energy-efficient synthetic routes for industrial applications that combine microwave heating and flow chemistry [176,177].

In conclusion, progress in green synthesis is closely associated with interdisciplinary approaches that integrate catalysis, nanotechnology, and sustainable energy management. Innovations in catalysts, solvent-free methodologies, and bio-inspired nanomaterials have demonstrated significant potential for reducing the environmental impact of chemical processes.

## 4. Functional Properties and Nutraceutical Potential

### 4.1. Conjugated Linoleic Acid (CLA)

CLA encompasses a group of naturally occurring positional and geometric isomers derived from LA that contain conjugated double bonds. Two isomers, namely c9,t11 and t10,c12, have sparked interest due to their anti-inflammatory and antioxidant activities. These isomers present anti-inflammatory properties through the regulation of production of cytokines (IL-6, TNF-α, IFN-γ, IL-1β), prostaglandins (PGE2), and leukotrienes (LTB4), which play an important role in the pathogenesis of many inflammation-mediated diseases [178,179,180]. Rheumatoid arthritis (RA) is one of the inflammatory diseases for which CLA has been shown to offer multiple benefits. RA is a recurring inflammatory disease that affects multiple systems and leads to cartilage destruction and synovial proliferation. Although its exact cause is still unknown, it is speculated that ROS production is involved in the pathogenesis of RA [181]. CLA is known to exert anti-inflammatory effects, which have been observed to help protect bones from damage. Moreover, CLA can increase bone mass, as demonstrated in several studies [182,183,184,185,186,187]. In other trials, CLA administration resulted in improved pain and morning stiffness status in patients suffering from RA after 12 weeks of treatment. In a study using a murine collagen-induced arthritis model, dietary CLA (c9,t11) was administered post-disease onset at dosages varying from 0.125% to 0.5% *w*/*w*. It was observed that even the lowest dosage of 0.125% CLA was sufficient to decrease plasma cytokines (IL-1β, TNF-α, IL-6) and reduce arthritis symptoms (paw swelling and clinical scores) by the end of the 84-day trial [188]. Similarly, it was demonstrated that 0.5% CLA (c9,t11) reduced arthritis symptoms through the reduction of IL-6 and IL-1β levels in mice comparable to a 1.5 mg/kg dose of celecoxib [189]. Muhlenbeck et al. also showed that both CLA isomers (trans-10, cis-12; and cis-9, trans-11) at 0.5% decreased the incidence of arthritis by 33% and 38%, respectively, and lowered IL-2 levels in mouse paws [190]. In a clinical trial conducted over a 3-month period on 22 participants, 2.5 g of daily supplementation with CLA resulted in lower levels of TNF-α, IL-1β, MMP-3, and CCP-A, indicating a beneficial effect in patients with active RA. Moreover, the treatment with CLA was well-tolerated, as only 2 participants reported side effects, which included flatulence [181].

Inflammatory bowel disease (IBD) is a chronic and recurrent inflammatory disease that affects the intestinal tract and comprises ulcerative colitis and Crohn’s disease [191]. CLA upregulates PPARγ and PPARδ in the colon and transcriptionally regulates the genes responsible for lipid metabolism and epithelial cell development, such as UCP1, UCP3, PGC-1α, and CD35 and Gob-4 and keratin 20, respectively. In an experimental model of colitis, CLA was shown to reduce inflammation caused by DSS and CD4^+^ by diminishing the production of TNF-α and suppressing NF-κB. At the same time, CLA boosted TGF-β1 levels, a cytokine considered to suppress excessive inflammation. However, these beneficial effects were lost when the PPARγ gene was removed from colon cells, suggesting that PPARγ is essential for CLA’s protective role in gut inflammation [192,193]. In a study, CLA production enhanced by *Bifidobacterium longum* CCFM681 ameliorated DSS-associated colitis by increasing MUC2, claudin-3, α-catenin 1, and ZO-1 (goblet cells involved in the protection of the intestinal mechanical barrier). The anti-inflammatory effects of CLA were also underlined by the inhibition of the TLR-4/NF-κB pathway, a reduction in IL-6 and TNF-α, and an increase in IL-10 levels [194]. Similarly, in another study conducted by Yang et al., it was proven that CLA-producing *Bifidobacterium breve* CCFM683 and 1% CLA-supplemented chow administration ameliorated DSS-induced colitis in mice by reducing pro-inflammatory cytokines (TNF-α, IL-6, IL-1β), upregulating levels of IL-10, MUC2, ZO-1, claudin-1, claudin-3, and E-cadherin. Additionally, CLA supplementation increased the *Lactobacillus* abundance, while decreasing the counts of harmful bacteria *Turicibacter*, *Allobaculum*, and *Sutterella* induced by DSS, and *B. breve* also decreased the Desulfovibrionaceae count, thereby restoring the microbial balance, which helped to decrease inflammation and improve colitis symptoms. The colonic CLA concentration increased to 3.23 mg/mL (direct supplementation) and 1.01 mg/mL (via *B. breve* CCFM 683), suggesting local CLA production contributed to the observed effects [195]. Daily administration of 6 g of CLA over 12 weeks in 13 patients led to notable immunomodulatory effects in mild to moderately active Crohn’s disease. CLA treatment reduced TNF-α, IFN-γ, and IL-17 counts by CD4+ and CD8+ T cells in peripheral blood, which caused a decrease in Crohn’s disease activity from 245 to 187, along with a significant improvement in patient-reported quality of life, reflected by an increase in the inflammatory bowel disease questionnaire score from 141 to 165. Additionally, CLA reduced the proliferation of CD3+ and CD4+ T cells, indicating a reduction in immune system activation, a response considered beneficial in the context of chronic intestinal inflammation [196].

Obesity and abnormal lipid profiles are key contributors to the development of hepatic steatosis, type 2 diabetes, cardiovascular diseases, and several types of cancers [197]. CLA has been increasingly recognized for its role in lipid regulation and fat metabolism due to its ability to decrease fat deposition, stimulate lipolysis through the oxidation of fatty acids in muscle cells and adipocytes, and enhance oxygen consumption alongside elevated energy usage [198,199,200]. In a study that was run over 9 weeks on high-fat diet-induced obesity rats, CLA administration resulted in decreased body mass and adipose tissue accumulation. These positive results were linked to the capacity of CLA to restore the intestinal microbiota composition (e.g., *Dubosiella*, *Faecalibaculum*, and *Bifidobacterium*), which further secretes short-chain fatty acids that participate in glycolipid metabolism through the activation of the INSR/IRS-2/AKT/GLUT4 pathway [201]. Similarly, in another study using a diet-induced obesity mouse model, high-purity CLA supplementation for 6 weeks led to a significant increase in intestinal levels of *Bacteroides* and decreased levels of *Firmicutes*. CLA also enriched butyrate- and acetate-producing bacteria, thereby enhancing microbial diversity, which was linked with improved metabolic health, serving as a potential food component that can be used to prevent obesity-related metabolic diseases [202]. He et al. showed that medium (600 mg/kg) and high (1800 mg/kg) doses of CLA reduced body weight, body fat mass (subcutaneous, perirenal, epididymal, and brown fat), and food intake in T2DM mice after 5 weeks. The medium-dose group experienced a reduction in body weight by 14.5%, while the high-dose group experienced a reduction of 41.1%. In addition, administration of the highest dosage of CLA and 100 mg/kg of t10,c12 CLA resulted in a significant decrease in triglycerides and total cholesterol, and the highest dosage of CLA and 100 mg/kg c9,t11 CLA treatment caused a reduction in the content of TG and TC. Moreover, CLA led to a decreased intestinal absorption of glucose and lipids, contributing to lower blood glucose and improved lipid profiles through the downregulation of the expression of glucose and lipid transporters GLUT2, GLUT5, and SLC5A1 and D36, ABCA1, and NPC1L1, respectively [203]. Supplementation with 3 g of CLA daily for 74 overweight/obese women over the course of 3 months led to significant reductions in the total body, visceral, android, and gynoid fat. In addition, there was a notable increase in lean body mass, implying an improved overall body composition compared to the placebo group [204].

**Figure 2:**
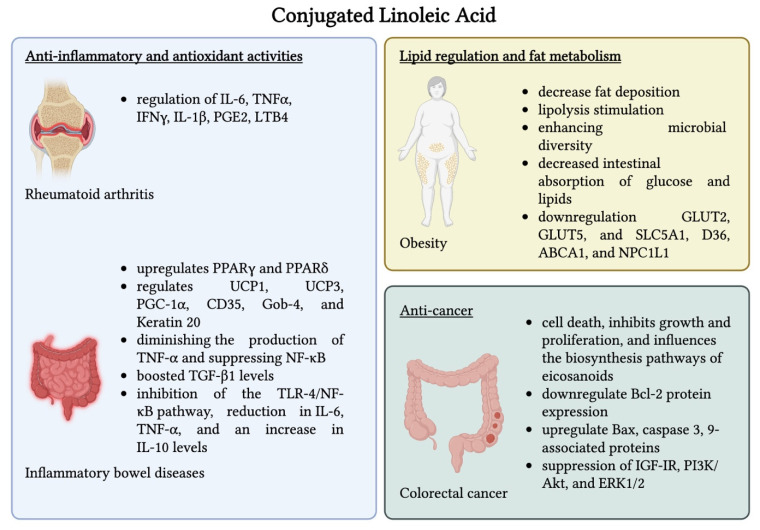
Graphic representation of the main biological effects of CLA as well as the associated mechanisms of action. Created in Biorender (https://BioRender.com). Stefania, D. (2025) https://BioRender.com/r7unm2c (accessed on 20 July 2025).

Colorectal cancer (CRC) is a significant global health burden, the 2nd deadliest cancer worldwide and the 3rd most frequently diagnosed, and it accounts for approximately 900,000 deaths annually [205]. Several in vitro and animal model studies reported that CLA administration induces cell death, inhibits growth and proliferation, and influences the biosynthesis pathways of eicosanoids in colon cancer cells [206,207,208,209,210,211]. The anti-cancer effect in colorectal carcinogenesis could be mediated through CLA’s ability to downregulate Bcl-2 protein expression and upregulate Bax and caspase 3- and 9-associated proteins, but also because it influences arachidonic acid metabolism [206,207,211,212]. Studies have also indicated that CLA demonstrates beneficial effects against CRC through the suppression of several key signaling molecules, such as IGF-IR, PI3K/Akt, and ERK1/2, as well as components of the APC-β, PPARδ, and PPARγ pathways [213,214,215]. A randomized placebo-controlled trial that was followed over a period of 6 weeks investigated the effects of daily 3 g CLA supplementation in 34 rectal cancer patients undergoing preoperative chemoradiotherapy. The outcomes showed that CLA administration resulted in lower IL-8 and carcinoembryonic antigen (CEA) levels compared to the control, as well as an increase in malondialdehyde (MDA), indicating lower oxidative stress. Although changes in leptin were not statistically significant, there was an upward trend in the group taking CLA. These results suggest that CLA may help modulate inflammation and oxidative stress during chemoradiotherapy, having potential implications for tumor activity and treatment tolerance [216]. Another trial that enrolled 31 rectal cancer patients undergoing preoperative chemoradiotherapy investigated the effects of daily 3 g CLA supplementation after 6 weeks. At the end of the trial, CLA increased levels of alpha-linolenic acid (ALA) and reduced the total *n*-6 PUFA level and the *n*-6/*n*-3 PUFA ratio. However, no statistically significant effects were observed on the overall lipid profile (TG, TC, LDL-C, and HDL-C) or on CEA levels when compared to the placebo. The increase in ALA was particularly relevant, as previous research had demonstrated that each 0.1% elevation in ALA levels in the bloodstream can lower the chance of developing colorectal cancer by 10% [205,217]. Figure 2 presents the main biological effects of CLA as well as the associated mechanisms of action.

#### Safety Considerations of CLA

In 2008, the FDA granted GRAS status to CLA isomers, approving their use in certain food products such as soy milk, meal replacement drinks and bars, dairy items, and fruit juices as long as the amount does not exceed 1.5 g per serving [218]. Similarly, EFSA evaluated CLA-rich oils, such as Clarinol^®^ (80% of the two CLA isomers c9,t11 and t10,c12, 1:1), and concluded that daily intakes of up to 3 g CLA (corresponding to 3.75 g Clarinol^®^) are safe for periods of up to 6 months [219]. Based on clinical evidence, daily supplementation with CLA appears to be safe at doses of up to 3.4 g for 2 years, or up to 6 g for 1 year [220]. A study conducted over 12 months in obese adults concluded that a daily dose of 6 g of CLA is safe and well-tolerated, with changes in glucose metabolism that were transient, and liver and insulin markers remained stable, contrasting with some previous animal studies that reported concerns about insulin resistance and liver toxicity [221,222,223]. However, high doses of CLA, particularly the t10,c12 isomer, have been linked to undesirable effects in mice, such as fatty liver, fat tissue atrophy, inflammation, and insulin resistance. Prolonged intake of t10,c12 CLA led to increased liver mass and TG accumulation due to stimulated lipogenesis, enhanced fatty acid oxidation, and impaired LDL secretion. In one study, a daily intake of 4.2 g of CLA effectively reduced fat mass in obese men but was also associated with impaired insulin sensitivity [220,224,225,226]. Other insights from clinical evidence reported that CLA administration (3.4–6.8 g from 12 weeks to 12 months) resulted in enhanced production of circulatory markers of oxidative stress, increased CRP, and digestive disturbances, such as dyspepsia, laxative effects, flatulence, gas bloating, loose stools, and nausea. Nonetheless, these effects were considered to be temporary and were regarded to be mild to moderate [227,228,229,230,231]. While CLA demonstrated promising benefits across a variety of health conditions, its effects can differ based on the isomer, dosage, duration, and individual response. Some adverse effects, although generally mild and transient, highlight the need for continued research and monitoring to fully grasp the long-term implications of CLA use to ensure its safe integration in both clinical and functional food applications.

### 4.2. 9- and 13-Hydroxyoctadecadienoic Acids (9- and 13-HODEs)

**Figure 3:**
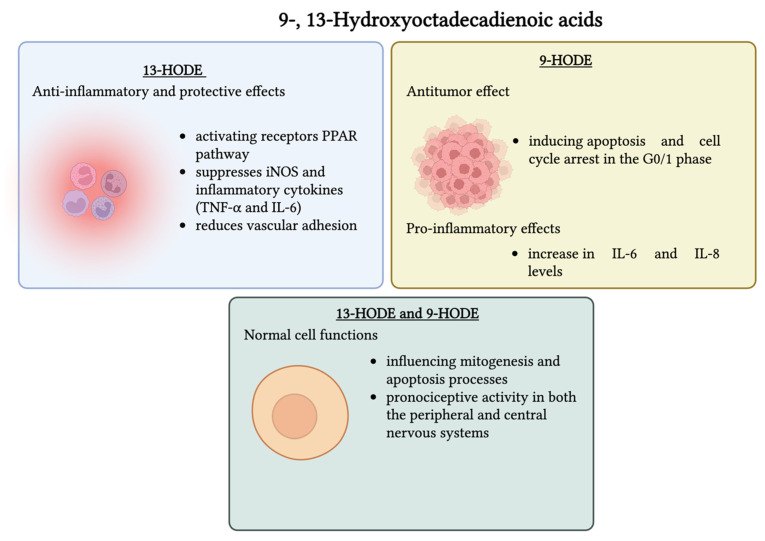
Graphic representation of the main biological effects of 13-HODE and 9-HODE, as well as their associated mechanisms of action. Created in BioRender. Stefania, D. (2025) https://BioRender.com/hmyrcv8 (accessed on 20 July 2025).

Such stable oxidation metabolites of LA as 9- and 13-HODEs are specialized pro-resolving mediators that have been shown to have distinct functions in modulating the transmission and inhibition of pain [232]. These oxidized derivatives of LA are involved in regulating inflammation, metabolic disorders, and cancer progression. Specifically, 13-HODE exerts anti-inflammatory and protective effects, mainly by activating the receptors of the PPAR pathway. In this way, it regulates lipid metabolism, suppresses iNOS and inflammatory cytokines (TNF-α and IL-6), and reduces vascular adhesion, all of which contribute to vascular health and anti-atherogenic properties. In addition, 13-HODE promotes the production of prostacyclin, a key inhibitor of platelet aggregation. In this way, it supports its anti-thrombotic role and potential benefit in cardiovascular disease. It also elevates cAMP levels, decreasing platelet and endothelial adhesion, and may exert extracellular vascular effects by lowering PGE_2_ production, thereby reflecting a protective role in vascular homeostasis. On the other hand, 9-HODE’s behavior depends on the context. Some studies indicate pro-inflammatory effects, for example, in the skin, where it causes an increase in IL-6 and IL-8 levels [233]. Both 9- and 13-HODEs maintain normal cell functions by influencing mitogenesis and apoptosis processes, often impaired in cancer cells. Hampel et al. found that 9-HODE had an antitumor effect in U937 cells by inducing apoptosis and cell cycle arrest in the G0/1 phase in both PPAR-dependent and independent pathways, while 13-HODE did not present anticancer effects on these cells [233,234,235]. Both 9- and 13-HODEs were previously associated with pro-nociceptive activity in both the peripheral and central nervous systems. Under inflammatory conditions (such as low pH), 9- and 13-HODEs may undergo further enzymatic conversion to bioactive epoxy–hydroxy or keto–epoxy derivatives, which were shown to sensitize nociceptive neurons and promote pain- or itch-like sensations [236]. In the circumstances of oxaliplatin-induced peripheral neuropathic pain, 9-HODE acted as a potent agonist of the G2A receptor. It sensitized the TRPV1 channel in a dose-dependent manner (100–200 nM) in dorsal root ganglion (DRG) neurons through a PKC-dependent signaling pathway. However, this pro-nociceptive effect was noted only under pathological conditions and not in healthy mice, indicating a context-specific action. On the other hand, 13-HODE did not induce sensitization under the same conditions, likely due to its weaker G2A receptor sensitivity or due to a need for much higher concentrations to achieve similar effects. An injection with 10 μM of 9-HODE into the paw of oxaliplatin-treated mice further enhanced mechanical hypersensitivity, whereas the same injection had no effect in naïve mice. These findings show that 9-HODE–G2A–TRPV1 signaling plays a role in amplifying pain during chemotherapy-induced nerve damage, pointing to this pathway as a potential therapeutic target [237]. In a murine model where inflammation was triggered with CFA, increased levels of 9- and 13-HODEs were detected in the paw tissue and DRG. When HODEs were neutralized with specific antibodies, it was noted that pain sensitivity significantly dropped, further confirming their involvement in inflammation [238]. Overall, 13-HODE is seen as anti-inflammatory, since it activates nuclear receptors such as PPARγ and PPARα, which regulate lipid metabolism in macrophages and help support the body’s natural recovery from inflammation. Moreover, it has been revealed that it has the ability to reduce platelet adhesion and lower the risk of thrombosis. Although 9-HODE also activates PPAR receptors, it can present a proinflammatory effect due to cytokine synthesis in RAW264.7 macrophages, and it can induce a stress response in the endoplasmic reticulum. Studies have proved that both 9- and 13-HODEs can trigger programmed cell death in macrophage cell lines and help regulate fat storage during the development of preadipocytes [239]. Together, these metabolites represent a double-edged sword, with pro-/anti-inflammatory effects based on the context and environmental stress. This underscores the importance of understanding the optimal balance between these HODEs, as their abundance may determine whether their effects are protective or harmful. Figure 3 presents the main biological effects of 13-HODE and 9-HODE as well as the associated mechanisms of action.

#### Safety Considerations of 9- and 13-HODEs

Metabolite 13-HODE normally supports vascular stability by inhibiting endothelial adhesion and promoting prostacyclin production. It may exert protective effects in the early stages of atherosclerosis by enhancing lipid clearance, inflammation control, and vascular stability through PPARγ activation. However, in situations where inflammation is present, its protective effects may be diminished, potentially contributing to vascular dysfunction. Meanwhile, 9-HODE is regarded generally as a pro-inflammatory metabolite. Nevertheless, both 9- and 13-HODEs can present harmful actions under conditions of prolonged oxidative stress or advanced atherosclerosis [234]. Further, 9-HODE induces intracellular calcium mobilization specifically in G2A-expressing cells in a dose-dependent manner, with half-maximal activation starting at approximately 2 μM and shifting to submicromolar concentrations when Gαqi is co-expressed [240]. Additionally, it was shown that 9-HODE is released under oxidative stress and is responsible for the production of IL-6 and IL-8 in human keratinocyte cells—an effect not observed with 13-HODE. Under pathological conditions, 9-HODE sensitized TRPV1 via G2A activation in a dose-dependent manner, promoting pain only in damaged tissue. In contrast, 13-HODE showed minimal effects [237]. Vangaveti et al. emphasized that 9- and 13-HODEs demonstrate pleiotropic, context- and dose-dependent effects. Under physiological conditions, 13-HODE normally acts in a tumor-suppressive manner by promoting apoptosis and reducing cell proliferation, for example, in colorectal and breast cancer cells, via downregulation of PPARδ signaling. Lower intracellular 13-HODE levels have been linked to poorer prognosis and enhanced tumor aggressiveness, especially in colon, breast, lung, pancreatic, and esophageal cancers. Similarly, 9-HODE shows dual behavior: while 9-HODE supports apoptotic pathways, 9-R-HODE has been implicated in promoting cell division and tumor progression [233]. Therefore, the biological impact of these two metabolites is highly dose- and context-dependent, and influenced by their enantiomeric form, reinforcing the need for caution on their therapeutic use.

### 4.3. Epoxygenated Fatty Acids (EpFAs)

**Figure 4:**
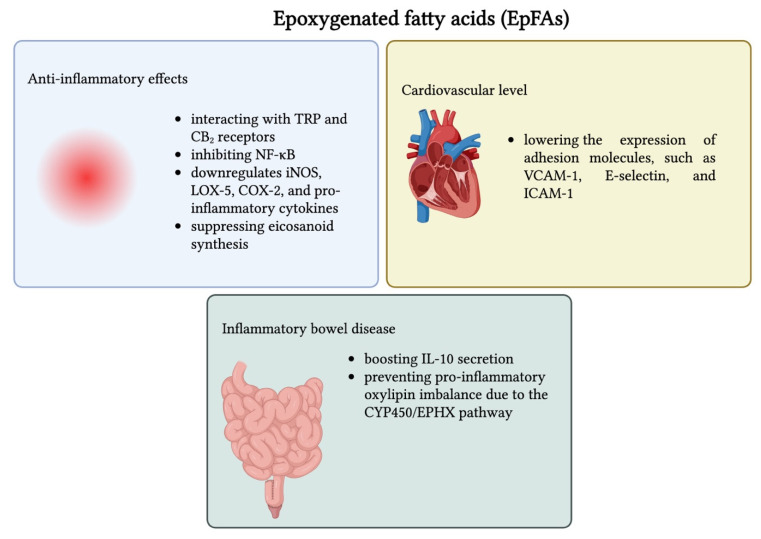
Graphic representation of the main biological effects of epoxygenated fatty acids (EpFAs), as well as their associated mechanisms of action. Created in BioRender. Stefania, D. (2025) https://BioRender.com/v426x8x (accessed on 20 July 2025).

Increased EpFA concentrations in the body are linked to beneficial and anti-inflammatory effects. They maintain homeostasis by stabilizing mitochondrial function, reducing oxidative and endoplasmic reticulum stress and inflammation, and promoting bronchodilatation. EpFAs demonstrate potent effects in inflammatory pain, mainly by interacting with TRP and CB_2_ receptors, inhibiting NF-κB, which downregulates iNOS, LOX-5, COX-2, and pro-inflammatory cytokines, and suppressing eicosanoid synthesis. In addition, EpFAs play a key role in protecting vascular health. By promoting vasodilatation through the activation of calcium-dependent potassium channels, they maintain endothelial function and reduce the risk of heart disease, high blood pressure, and stroke [241,242,243]. Dysregulation of EpFA metabolism has been observed to contribute to pathological conditions such as preeclampsia by impairing placental angiogenesis and exacerbating hypertension [244]. At the cardiovascular level, EpFAs reduce inflammation by lowering the expression of adhesion molecules, such as VCAM-1, E-selectin, and ICAM-1 [245]. Soluble epoxide hydrolase inhibitors (sEHIs) work by preventing the breakdown of EpFAs, leading to their accumulation in tissues. Interestingly, while originally studied for their role in inflammation, sEHIs were found to significantly reduce neuropathic pain, a discovery made incidentally while studying inflammatory pain models. Compared to standard treatments such as gabapentin, sEHIs demonstrated greater analgesia and better pharmacokinetic profiles in an experimental model of type 1 diabetes. Consequently, enhancing endogenous EpFA levels through sEH inhibition offers a promising therapeutic strategy for treating chronic and neuropathic pain [242]. EpFAs’ implications in IBD have also been demonstrated by maintaining anti-inflammatory signaling in macrophages, boosting IL-10 secretion, and preventing pro-inflammatory oxylipin imbalance, primarily due to the CYP450/EPHX pathway [246]. In numerous studies, the inhibition of sEH resulted in strong anti-inflammatory effects in IBD and ulcer models by keeping EpFA levels increased [247,248,249]. In IL-10-deficient mice, limiting sEH activity decreased ulcers, tissue inflammation, and inflammatory cytokine expression (IFN-γ, TNF-α, MCP-1, VCAM-1), along with the downregulation of phosphorylated NF-κB. EpFAs also restored the balance of oxylipins and lowered pro-inflammatory metabolites LTB4 and 5-HETE [247]. Furthermore, sEH deletion lowered the risk of developing adenocarcinoma from IBD [249]. In NSAID-induced intestinal ulcers, sEHI treatment protected the gut due to elevated EPFA levels, suggesting they mediate mucosal healing and inflammation resolution [250]. Figure 4 presents the main biological effects of epoxygenated fatty acids (EpFAs), as well as the associated mechanisms of action.

#### Safety Considerations of EpFAs

Multiple clinical trials with small-molecule sEHIs have demonstrated favorable safety profiles in humans. For example, AR9281 was well-tolerated at doses of up to 1.2 g/day for 7 days [251]. Similarly, other clinical trials were launched to assess the safety of GSK2256294 in healthy individuals, as well as in obese smokers, for endothelial function. Participants received daily oral doses ranging from 2 to 20 mg over a 14-day period. Overall, the compound was well-tolerated, with side effects that were categorized as mild to moderate (e.g., transient headaches, contact dermatitis). In addition, headaches appeared at similar rates in both the treatment and the placebo groups, a sign that they were not related to the drug [252]. Icosabutate (EpFA mimic) was evaluated in 87 patients suffering from severe hypertriglyceridemia who received 600 mg daily for 12 weeks. It proved to be generally safe, with most effects being mild, such as slight gastrointestinal disturbances (diarrhea, abdominal discomfort, nausea), minor skin reactions (pruritus, erythema), or musculoskeletal disorders (back pain, myalgia). In 7 patients, some infections occurred, including nasopharyngitis, urinary tract infection, and sinusitis. Importantly, no serious drug-related adverse events were reported, and no clinically significant changes were observed in laboratory parameters, vital signs, or ECGs [253]. In a clinical trial, another EpFA mimic (icosapent ethyl, Vascepa) was evaluated in 8179 patients over a median of 4.9 years at a dose of 4 g per day. It proved to have a good safety profile, with adverse events comparable to a placebo. However, there was a slight increase in atrial fibrillation from 2.1% to 3.1% and serious bleeding events from 2.1% to 2.7%, though no fatal bleeding occurred. Vascepa has since been approved by the FDA to reduce cardiovascular risk in high-risk adults with elevated triglycerides [254,255]. Overall, clinical trials have consistently shown that both sEHIs and EpFA mimics have favorable safety profiles, with only mild to moderate adverse effects observed. These findings support their continued development as promising therapeutic agents for various diseases.

### 4.4. Oxo-Fatty Acids (Oxo-FAs)

**Figure 5:**
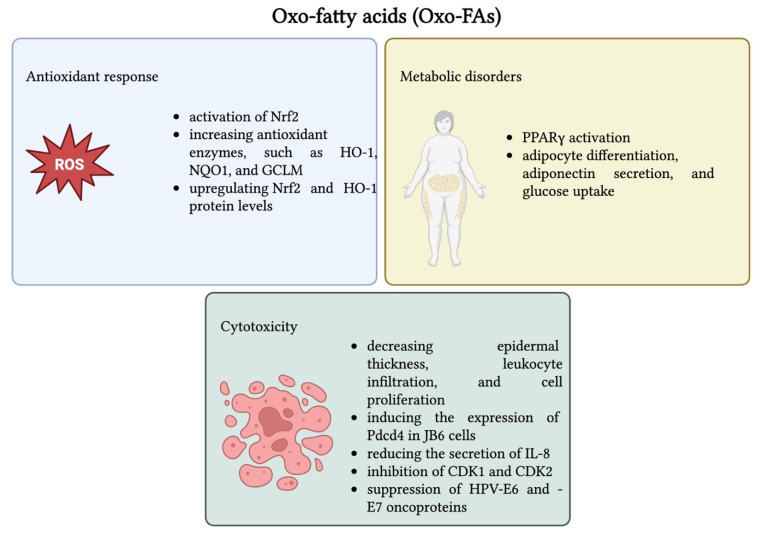
Graphic representation of the main biological effects of oxo-fatty acids (oxo-FAs), as well as their associated mechanisms of action. Created in BioRender. Stefania, D. (2025) https://BioRender.com/w3r5zhl.

Oxo-FAs regulate several signaling pathways, including PKC-β, NF-κB, and PPAR, which explain their diverse biological activities, such as modulating inflammation, regulating cell cycle, and influencing programmed cell death [256]. In an in vitro study, 30 µM of 10-oxo-trans-11-octadecenoic acid was found to activate the Nrf2–ARE pathway, offering a protective effect on the HepG2 liver cells against the cytotoxicity induced by H_2_O_2_ by increasing Nrf2 protein levels and increasing antioxidant enzymes, such as HO-1, NQO1, and GCLM. The cytoprotective effects against oxidative stress were also observed in vivo, with a dose as low as 3 mg/mouse significantly upregulating Nrf2 and HO-1 protein levels [257]. Goto et al. demonstrated that among the oxo-linoleats produced by gut lactic acid bacteria, both 10-oxo-12(Z)-octadecenoic acid (KetoA) and 10-oxo-11(E)-octadecenoic acid (KetoC) strongly activated PPARγ at 30 µM. However, KetoA was more potent at 100 µM in NIH-3T3 cells. Additionally, KetoA promoted adipocyte differentiation, adiponectin secretion, and glucose uptake in 3T3-L1 cells at 30 µM over 8 days, making it a promising candidate against metabolic disorders such as obesity and type 2 diabetes [258]. In a two-stage carcinogenesis model, pretreatment with 1600 nmol of 13-HOA resulted in a 95% reduction in TPA-induced ear inflammation. This was accompanied by a 40% decrease in tumor incidence and a 64% drop in tumors per mouse. Furthermore, it decreased epidermal thickness, leukocyte infiltration, and cell proliferation, as observed after the histological examination. Additionally, at concentrations of 8–40 μM, 13-HOA significantly suppressed TPA-induced anchorage-independent growth in JB6 epidermal cells by 70–100%, and at 40 μM, it inhibited AP-1 activity. Notably, post-treatment with 13-HOA at 40 μM and 1600 nmol induced the expression of Pdcd4 in JB6 cells and mouse skin, which is particularly important because it suppresses tumor growth [259]. Further, 13-oxo-ODE is a ligand endogenously produced for PPARγ in colonic epithelial cells by the oxidation of LA. When it was applied for 24 h in HT-29 cells at concentrations ranging from 3.12 to 100 μM, it reduced the secretion of IL-8 in a dose-dependent manner. The pharmacological effect was correlated to its direct binding to PPARγ, making it a potential therapeutic agent in IBD [260]. In a study conducted over a period of 72 h, some saturated oxo-fatty acids (SOFAs) were evaluated regarding their cytotoxic effects against various human cancer cell lines at concentrations of 10–75 μM; 5-, 6-, 7-, and 8-OSA were shown to exert cytotoxic effects in A549 cells, having IC_50_ values of 49, 26, 28, and 37 μM. Although some inhibitory activity was observed in Caco-2 and SF268 cells, the effect was cell-specific and less pronounced. Notably, 6-OSA was tested for toxicity on normal human umbilical vein endothelial cells (HUVECs) and proved to have selective activity, showing no cytotoxic effects at concentrations of up to 75 μM [261]. Further, 9-oxo-ODAs derived from eggplant calyx have shown promising anticancer effects in HPV-positive cervical cancer cells (HeLa, SiHa). They induced apoptosis in a concentration-dependent manner from dosages varying from 25–50 μM over 3–5 days through the inhibition of CDK1 and CDK2 and by the suppression of HPV-E6 and -E7 oncoproteins [262]. Figure 5 presents the main biological effects of oxo-fatty acids (oxo-FAs), as well as the associated mechanisms of action.

#### Safety Considerations of Oxo-FAs

Oxo-FAs are highly reactive and have been associated with toxic effects, such as 9-oxo-9:0-FA, which was implicated for inducing hepatic lipid peroxidation [263]. In spite of that, not all 9-oxo-FAs share the same risks. For instance, 9-oxo-ODA found in tomato fruit reduced intracellular triglyceride accumulation in hepatocytes and upregulated PPARα target genes in mice at 10 and 20 μM, with the effect rising with the increase in dosage [264]. In another study, 9-oxo-ODA showed a selective cytotoxic effect towards normal human peritoneal mesothelial cells compared to HPV–cervical cancer cell lines, suggesting a favorable safety profile and potential therapeutic development against HPV-related cancers [262]. In its turn, 13-oxo-ODA extracted from tomato juice has demonstrated PPARα-mediated benefits without observable toxicity. In an in vivo study, supplementation with 13-oxo-ODA significantly improved lipid and glucose metabolism, reduced hepatic and skeletal muscle triglyceride accumulation, and enhanced fatty acid oxidation, all without altering body weight or the liver of the mice in a dose-dependent manner (0.02% and 0.05% *w*/*w*) [265]. However, 9- and 13-oxo-ODAs show a dual nature sometimes—they can act as bioactive markers and potential mediators of liver injury in type 2 diabetes in obese adolescents, especially in the context of hepatic steatosis and PNPLA3 genetic susceptibility. While these compounds have been linked to oxidative stress and impaired insulin secretion, they also exhibit therapeutic potential, as demonstrated in other studies [233,266,267,268]. KetoA and KetoC, derived from *Lactobacillus plantarum*, demonstrated promising protective effects against the progression of nonalcoholic steatohepatitis in mice without causing toxicity at 0.1% *w*/*w* after 3 weeks. Both compounds showed no adverse effects in body or organ weights, liver enzymes, or other metabolic parameters, indicating a favorable safety profile. On the contrary, KetoC remarkably increased HDL cholesterol, while KetoA reduced inflammation markers such as TNF-α and prevented the development of bridging fibrosis [269]. Despite their promising metabolic benefits, oxo-FAs present complex and context-dependent biological effects. For this reason, more in vivo and clinical research is needed to clarify their safety as potential nutraceuticals.

### 4.5. Linoleic Acid-Derived Esters

**Figure 6:**
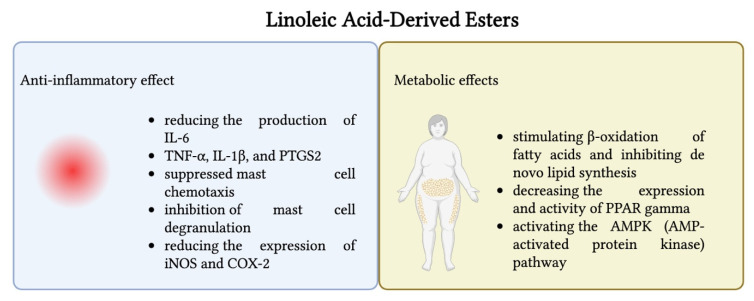
Graphic representation of the main biological effects of linoleic acid-derived esters, as well as their associated mechanisms of action. Created in BioRender. Stefania, D. (2025) https://BioRender.com/oddz0k3.

Linoleic acid-derived esters, such as 13-DHAHLA, have emerged as bioactive lipids with remarkable anti-inflammatory and metabolic effects. In an in vitro study, 10 μmol/L of 13-DHAHLA demonstrated a significant capacity to reduce inflammation in LPS-induced RAW 264.7 macrophages, reducing the production of IL-6, TNF-α, IL-1β, and PTGS2. These biological effects were observed even at concentrations as low as 10 nmol/L [270]. Paluchova et al. showed that both 13(S)- and 13(R)-DHAHLA isomers obtained from triacylglycerol-rich oils of marine origin showed anti-inflammatory activity in bone marrow-derived mast cells from mice (BMMCs), which play a central role in allergic and inflammatory responses [271,272]. Treatment with either isomer at a concentration of 1 μM effectively suppressed mast cell chemotaxis in response to antigens and PGE2. Moreover, pretreatment with 4 μM of 13-DHAHLA isomers led to the inhibition of mast cell degranulation, as indicated by the decreased release of β-glucuronidase [271]. Further, 13-LAHLA, the most abundant FAHFA (fatty acid esters of hydroxy-fatty acid) found in oat oil, was discovered to inhibit mRNA levels of IL-6 and IL-1β in LPS-treated RAW 264.7 macrophages. Additionally, it also reduced the expression of iNOS and COX-2 by 50% under the same conditions at 10 μM. Interestingly, it appears that its activity is specific to inflammatory activation, as its effect was absent on IL-6 levels in LPS-unstimulated cells [273]. It has been shown that methyl and ethyl ester derivatives of linoleic acid have a complex metabolic profile, which may be useful for the prevention and management of metabolic disorders. These compounds favorably influence lipid metabolism by stimulating β-oxidation of fatty acids and inhibiting de novo lipid synthesis, a phenomenon characterized by a negative regulation of the expression of enzymes involved in lipogenesis [274,275]. As well as exerting antiadipogenic effects, these esters decrease the expression and activity of PPAR gamma, thus inhibiting the differentiation of preadipocytes [276]. Additionally, they have the ability to activate the AMPK (AMP-activated protein kinase) pathway, resulting in improved glucose homeostasis and insulin sensitivity [277]. These mechanisms make linoleic acid ester derivatives promising bioactive compounds for the formulation of functional food products and nutritional interventions intended to support metabolic health. Figure 6 presents the main biological effects of linoleic acid-derived esters, as well as the associated mechanisms of action.

#### Safety Considerations of Linoleic Acid-Derived Esters

Safety studies on the linoleic acid-derived esters, DHAHLA and LAHLA, are limited. Esters 9- and 13-DHAHLA are synthesized in adipose tissue after *n*-3 PUFA supplementation. They exhibit anti-inflammatory and pro-resolving effects without inducing cytotoxicity in vitro. It is plausible that DHAHLA may present beneficial anti-inflammatory effects in both human and murine white adipose tissue, as has already been discovered for other DHA products [270]. An in vivo study reported no adverse effects after repeated administration of TGA-rich marine oils containing DHA (up to 12 µg over 8 days), which elevated 13-DHAHLA levels. Additionally, the anti-inflammatory effects of these esters suggest they are beneficial rather than harmful; however, clinical studies are required to fully establish their safety [271]. In a study conducted by Brezinova et al., 13-DHAHLA was detected in human milk in one mother who had reported omega-3 PUFA supplementation during pregnancy. No adverse effects were associated with its presence [278]. Further, 13-LAHLA is a naturally occurring anti-inflammatory lipid in plants, mice, and humans. It proved to be non-toxic to RAW 264.7 macrophages [273]. However, more toxicological studies are needed to further assess the esters’ safety profiles.

## 5. Valorization in the Food Industry

Green synthesis of linoleic acid derivatives represents a significant development in the field. It is aligned with the current trends that promote environmental protection, consumer welfare, and the use of sustainable technologies [84]. As previously discussed, green synthesis is capable of producing derivatives with high purity, increased functionality, and reduced environmental impact [279]. Due to their unique properties, including antioxidant and antimicrobial properties, these compounds are ideal agents for food preservation and shelf life extension. It is also important to note that the use of linoleic acid derivatives in the food industry contributes to the nutritional improvement of food, since these derivatives have been associated with several important health benefits, such as modulating lipid metabolism and reducing inflammation [85]. As a result, adding them to functional foods improves the quality of the product while reducing the need for harmful additives [280]. Research and optimization of ecological synthesis protocols will enable the further refinement of these compounds in the evolving landscape of food industry innovation, thus consolidating their role as sustainable and efficient additives.

### 5.1. Incorporation in Functional Foods and Beverages

The incorporation of linoleic acid derivatives into functional food and beverage systems represents an integrated approach that integrates lipid chemistry, food technology, colloid science, and nutritional biochemistry. In view of their unique physicochemical and bioactive properties, these derivatives require innovative strategies for their incorporation, stabilization, and targeted delivery to ensure both their functional effectiveness and their sensory acceptability [281,282].

#### 5.1.1. Technological Considerations for Incorporation

The successful incorporation of linoleic acid derivatives, such as conjugated linoleic acid (CLA), hydroxylinoleic acids (HODEs), oxylipins, and epoxy-linoleates, depends on several factors: oxidative stability, polarity, volatility, solubility in aqueous or lipid systems, interaction with other matrix compounds (e.g., proteins, polysaccharides), and gastrointestinal degradation susceptibility [283,284,285]. These factors vary significantly between different food categories, requiring tailored approaches, such as (i) micro- and nanoencapsulation in biopolymer matrices (e.g., alginate, whey proteins, chitosan), solid lipid nanoparticles (SLNs), nanostructured lipid carriers (NLCs) or liposomes; (ii) complexation with cyclodextrins to improve aqueous solubility and reduce the impact on odor or taste; (iii) co-formulation with synergistic bioactive compounds, such as polyphenols or tocopherols, for increased oxidative stability and synergistic health benefits; (iv) layer-by-layer assembly of multilamellar emulsions to prevent oxidation and facilitate controlled release in the gastrointestinal tract [286,287].

#### 5.1.2. Dairy-Based Functional Products

A lipid-based food system, such as dairy products (milk, yogurt, cheese), offers optimal matrices for the delivery of linoleic acid derivatives because they contain natural lipids and emulsified proteins, as well as the capacity to buffer these products. The high affinity of hydrophobic LA derivatives (CLA isomers) for casein micelles and milk fat globule membranes facilitates their incorporation without significant phase separation [288,289].

A promising approach involves the production of conjugated linoleic acid (CLA) in situ using fermentation. Co-fermentation of milk with *Lactobacillus plantarum* and *Propionibacterium freudenreichii* results in the direct synthesis of CLA in the food matrix, which reduces oxidative degradation and enhances bioavailability [94]. Another effective strategy is the encapsulation of the derivatives in milk proteins. By using nanoparticles based on whey protein isolate (WPI) and caseinate, it is possible to increase stability during thermal processes such as pasteurization and storage while maintaining bioaccessibility [290].

In the food matrix, protein–fat interactions play an important role in protecting linoleic acid derivatives from the action of digestive enzymes, particularly gastrointestinal lipases. This phenomenon favors a controlled release of the active compounds in the intestines [291]. Furthermore, the micellar behavior of casein-linked derivatives may change under gastric conditions, contributing to more efficient absorption in the intestinal mucosa [292,293].

#### 5.1.3. Plant-Based Emulsified Systems

As the trend toward plant-based alternatives grows, fortifying emulsified beverages such as soy, oat, almond, or pea milk with linoleic acid (LA) derivatives is becoming increasingly important [294,295]. It is, however, necessary to develop synthetic or recombinant emulsifiers as well as stabilizing agents to ensure the stability and productivity of these systems in the absence of native animal milk proteins [296].

Among the delivery systems, Pickering emulsions stabilized with plant-derived nanoparticles (such as cellulose nanocrystals or zein particles) have proven effective, offering superior oxidation resistance and high thermal stability [297]. Furthermore, technologies such as high-pressure homogenization and ultrasonic emulsification can produce oil-in-water nanoemulsions with dimensions below 200 nm, improving the clarity of the final product and increasing the bioavailability of LA derivatives [298,299].

In spite of this, plant-based systems present a number of technological challenges. These beverages contain high levels of polyphenols, which may lead to redox interactions with LA derivatives and oxidative instability [300]. Moreover, variations in pH and ionic strength in the plant matrix could compromise the stability of emulsions if appropriate buffers are not provided [301,302].

An example of such a nanoemulsion is a pea protein-stabilized nanoemulsion containing structured lipids derived from CLA. In in vitro digestion models using Caco-2 cell lines, the product demonstrated a physical stability of over 95% over 90 days at 4 °C [303].

#### 5.1.4. Functional Bakery and Snack Products

Incorporating linoleic acid (LA) derivatives into solid and semi-solid food matrices, including bakery products, nutrition bars, extrudates, and expanded snacks, presents significant thermal and oxidative challenges, primarily because of the high processing temperatures (≥180 °C) and prolonged exposure to oxygen during the technological process [304,305].

Advanced technological strategies have been developed to address these limitations, including the use of solid lipid microparticles (SLMs) and waxy carriers with high melting points, such as carnauba wax or beeswax, which provide a high degree of thermal and oxidative protection during baking of LA derivatives [306,307]. Protein–lipid conjugates obtained by Maillard-type reactions between whey proteins and glucose, which can function simultaneously as protective agents and natural antioxidants, were also highlighted [308].

Thermal processes can induce partial oxidation of LA derivatives, resulting in the formation of bioactive oxylipins directly in the food matrix [309]. This method of in situ metabolite generation may be exploited functionally, provided that the process is carefully managed to produce the desired physiological effects. Additionally, delayed-release systems based on resistant starch or dietary fibers have been proposed for facilitating the colonic delivery of LA metabolites that may have potential anti-inflammatory or prebiotic properties [310].

Products fortified with encapsulated CLA have demonstrated the applicability of these strategies, including functional biscuits that retained more than 70% of their original isomer content after baking, as well as improved postprandial lipid profiles in murine test models [311].

#### 5.1.5. Functional Beverages: Aqueous and Carbonated Systems

Aqueous functional beverages, particularly those based on linoleic acid (LA), present a unique technological challenge due to their hydrophobic characteristics and high volatility. In order to effectively integrate these bioactive compounds into such systems, emulsion engineering, colloidal stabilization, and sensory masking of unpleasant tastes and odors are required [312,313,314].

Innovations in delivery platforms have provided solutions to these challenges. Cyclodextrin inclusion complexes, especially those based on β- and γ-cyclodextrin, have proven effective in encapsulating compounds such as CLA and oxylipins, providing a significant improvement in water solubility and a reduction in the perception of undesirable flavors [315,316]. As well, self-emulsifying delivery systems (SEDS), which include surfactants and co-solvents, produce microemulsions upon ingestion, facilitating the solubilization of hydrophobic compounds [317,318].

A key issue is the compatibility between the liquid matrix and the delivery system. For carbonated beverages, emulsion stability can be maintained by using non-ionic surfactants and fine pH adjustments, preventing phase separation and creaming phenomena [319]. For low-solid beverages such as functional waters and isotonic beverages, nanoemulsions are generally acceptable at low concentrations (up to 0.5%) without affecting their clarity or transparency [320].

Long-term stability of LA derivatives in aqueous and oxidizing environments is a major concern, requiring additional protective measures. The co-formulation of natural antioxidants, such as ascorbic acid or rosmarinic acid, can significantly extend the shelf life of products by limiting oxidative reactions. In addition, it is necessary to use opaque and oxygen barrier packaging, such as multilayer PET bottles with oxygen absorbers, in order to prevent light- and air-induced degradation, thereby ensuring the functional and sensory integrity of the product [321].

#### 5.1.6. Nutraceutical Powders and Meal Replacements

Nutrition bars, protein shakes, or powdered supplements with extended shelf life are practical and effective ways to administer linoleic acid (LA) derivatives to patients and athletes. In these forms, precise dosing is possible, the product is more stable under ambient conditions, and they are easy to integrate into a personal diet [322,323].

Converting LA derivatives into dry solid forms involves advanced encapsulation and drying technologies [324]. Processes such as spray-drying, freeze-drying, or fluidized coating allow the conversion of derivatives into stable powder forms using carriers such as maltodextrin, inulin, or resistant dextrins. In addition, coacervation techniques, in particular, those based on gelatin–gum arabic systems, have been exploited to obtain microcapsules with high loading efficiency and minimal loss of the active substance [325]. To improve bioavailability, some powder systems use enteric coatings or pH-sensitive polymers, which ensure controlled release of the compounds in the intestines, thus avoiding acid degradation in the stomach [326,327]. Additionally, the incorporation of bioactive compounds that act as bio-potentiators, such as piperine or quercetin, can enhance intestinal absorption of LA derivatives by modulating membrane transporters or inhibiting efflux pumps, thus optimizing the biological utilization of these functional lipids [328].

The clinical relevance of these formulations has been proven in a 12-week randomized controlled trial in which a powdered medical food enriched with 13-HODE and CLA induced favorable changes in eicosanoid profiles and improved inflammatory markers in patients diagnosed with metabolic syndrome [329,330].

#### 5.1.7. Integration into Personalized Nutrition Paradigms

In recent years, advances in microbiome sequencing and lipidomics have allowed for the precise stratification of individuals according to their response to functional foods containing linoleic acid derivatives [331]. Using this approach, nutritional interventions can be personalized by taking into account the enterotypic microbiota type, fecal lipidome signature, and host genetic polymorphisms controlling fatty acid transport or PPAR receptor isoform expression. As a result of the use of these molecular diagnostic tools, precision nutrition is becoming increasingly possible, where functional foods can be tailored to the microbiota profile of each individual in order to maximize their therapeutic and preventive efficacy [332].

### 5.2. Role as Food Preservatives and Stabilizers

The utility of linoleic acid (LA) and its ecologically synthesized derivatives as natural preservatives and stabilizers in functional foods and beverages goes far beyond their intrinsic nutritional value [94]. These compounds exhibit antioxidant, antimicrobial, and emulsion-stabilizing properties that can be exploited to improve the shelf life, microbiological safety, and physicochemical stability of food systems [323,333,334]. In particular, derivatives synthesized using green chemistry methods—such as conjugated, hydroxy-, epoxy-, and oxo-derivatives of linoleic acid—exhibit superior functional properties due to structural modifications that affect the polarity, electron density and chemical reactivity of the molecules, giving them increased technological and bioactive potential in the context of modern functional foods [335,336].

#### 5.2.1. Antioxidant Activity and Lipid Oxidation Inhibition

Foods containing high amounts of unsaturated fats are at greater risk of deterioration due to lipid oxidation, which can result in rancidity, undesirable flavors and odors, loss of nutritional value, and the generation of toxic secondary compounds [337]. In this regard, linoleic acid (LA) and certain oxidized or conjugated derivatives have been shown to possess antioxidant properties. The nature of the functional groups and the oxidation state of the molecule determine the ability of these derivatives to interrupt radical chain reactions or complex pro-oxidizing metal ions [233,338,339].

Derivatives of LA, particularly hydroxy- and oxo-derivatives, have been shown to neutralize peroxyl radicals and inhibit lipid peroxidation in both bulk oils and emulsions. As an example of this ability, hydroxy-octadecadienoic acids (13-HODE and 9-HODE) can be cited. Hydroxyl groups at the C9 or C13 positions provide reactive centers for hydrogen atom donation, thus interrupting the propagation phase of radical reactions [340].

In the field of antioxidants, conjugated linoleic acid (CLA) is of particular interest because of its isomer effects. According to recent research, CLA exerts indirect antioxidant effects by activating endogenous cellular defense mechanisms, particularly the Nrf2–ARE (antioxidant response element) pathway [341]. The presence of CLA in emulsified protein–lipid matrices contributes to the reduction of protein carbonylation and the reduction of lipid hydroperoxide accumulation. In terms of the mechanism, CLA functions as a preferential substrate in oxidative reactions, protecting other critical components of the food matrix. Moreover, it has been observed that it stimulates the expression of antioxidant enzymes within the cell, including glutathione peroxidase and catalase. As a result of this stimulation, the food product in which it is included has greater oxidative stability [342].

#### 5.2.2. Antimicrobial Effects and Microbiological Preservation

A broad spectrum of antimicrobial activity has been demonstrated against Gram-positive bacteria and some fungal species by LA derivatives [343,344]. Among the mechanisms involved in the antimicrobial effect are disruption of the microbial membrane integrity, interference with fatty acid biosynthesis, and the production of reactive oxygen species (ROS). There is evidence that these processes cause significant metabolic imbalance, which in turn affects the viability and functionality of pathogens and spoilage agents [345].

Depending on the derivative, the spectrum of antimicrobial action differs. CLA isomers have shown efficacy against food pathogens such as *Listeria monocytogenes*, *Bacillus cereus*, *Staphylococcus aureus*, and *Salmonella enterica* [346,347]. *Zygosaccharomyces bailii* and *Aspergillus niger*, molds of industrial importance, have been shown to be inhibited by hydroxylinoleate derivatives [344]. In particular, epoxy-linoleic acids display significant antifungal properties due to their electrophilic nature. The reactivity of these molecules allows them to interact covalently with thiol-containing proteins in fungal membranes, which results in structural destabilization of the cell [348].

The use of conjugated linoleic acid (CLA) isomers in hamburger patties, as well as in edible whey protein isolate (WPI) films, is a definitive illustration of their utility. In a study evaluating the quality of cooked, vacuum-packed hamburger patties stored at 4 °C for 60 days, CLA was found to significantly reduce lipid oxidation processes, as indicated by lower thiobarbituric acid reactive substance (TBARS) values and a decrease in total mesophilic aerobic bacteria (TMAB). Additionally, CLA-treated samples showed favorable changes in pH and color parameters. Based on the results, the integration of CLA into meat products and edible packaging materials could be an effective method for extending the shelf life under refrigerated conditions by retarding oxidative processes and preventing microbiological spoilage [349].

In terms of their mechanism of action, LA derivatives possess multifactorial antimicrobial activity. In the first instance, LA derivatives can intercalate into the lipid bilayer of the microbial cell membrane, causing an increase in membrane fluidity and permeability, resulting in the leakage of cytoplasmic contents [345]. Second, certain compounds, especially CLA, have been shown to inhibit enoyl-ACP reductase (FabI), which participates in bacterial fatty acid biosynthesis, thereby affecting membrane synthesis. Furthermore, epoxidized and oxo-derivatives of LA promote oxidative stress by generating intracellular ROS. This disrupts the homeostasis of microorganisms and contributes to their lethality [350].

#### 5.2.3. Emulsifying and Physical Stabilization Properties

In addition to their biochemical functions in food preservation, linoleic acid (LA) derivatives contribute significantly to the stability of multiphase systems, including oil-in-water (O/W) emulsions. A number of these compounds exert favorable effects on interfacial tension and interact with proteins and polysaccharides at the phase interface, facilitating formation of interfacial layers and stabilization at the phase interface, thereby preventing lipid droplets from aggregating or coalescing [333].

The lipids formed by enzymatic esterification of linoleic acid with medium-chain triglycerides (MCTs) are amphiphilic, making them suitable for use as co-surfactants. By arranging themselves on the surface of oil droplets, these molecules increase the interfacial coverage and reduce the tendency to coalesce [351,352]. As an example, the use of a structured lipid based on CLA and monoglycerides in a dual emulsion system (water-in-oil-in-water, W/O/W) enhanced phase separation resistance and lipophilic bioactive compound encapsulation efficiency over 30 days of storage [353].

Furthermore, LA hydroxy derivatives have a strong affinity for food biopolymers, such as whey proteins, soy proteins, or pectin, with which they form hydrogen bonds and hydrophobic interactions. The interaction between these molecules strengthens the interfacial layer and enhances the physical stability of emulsions [354,355]. As a result of their ability to modify the secondary structures of proteins adsorbed at the oil–water interface, these derivatives can modify the interfacial behavior of proteins. For example, the co-adsorption of 13-HODE with β-lactoglobulin leads to the reorganization of its structure at the interface, resulting in increased viscoelasticity of the interfacial layer and preventing flocculation in acidic beverages. Thus, LA derivatives play a dual role—both functional and technological—in stabilizing dispersed food systems [323,356,357].

#### 5.2.4. Synergistic Combinations with Other Natural Preservatives

The preservative activity of linoleic acid (LA) derivatives can be enhanced significantly by combining them with essential oils, polyphenols, or organic acids [343]. In addition to increasing the effectiveness of inhibiting oxidation and microbial growth, these synergistic interactions result in a reduction in the doses required to achieve the desired effects, contributing to the preservation of sensory qualities and greater acceptance by consumers [358].

The conjugated linoleic acid (CLA) and carvacrol association, for example, results in a dual effect on microbial cell membranes as a result of physical destabilization of the membrane and collapse of the proton motive force, which enhances the efficacy of the antibacterial compound against spoilage microorganisms in fleshy matrices [345,359]. Ascorbic acid and 13-HODE work together synergistically to reduce lipid oxidation and microbial growth in high-fat emulsified dairy systems. This contributes to a longer shelf life for the product. Additionally, the interaction between CLA and tannic acid results in the formation of lipid–polyphenol complexes with prolonged antioxidant activity that are maintained during storage [360,361].

These synergistic effects allow the optimization of functional food formulations by lowering the required concentrations of each active compound, reducing the impact on the sensory profile of the product, and facilitating the development of sustainable preservative solutions compatible with the current clean label requirements [362,363].

#### 5.2.5. Applications in Packaging and Edible Films

Derivatives of linoleic acid can be used not only in direct food formulations, but also in the development of bioactive packaging materials, which contribute to the passive preservation of food products by controlling release mechanisms. As these compounds are incorporated into biodegradable matrices, it is possible to produce functional packaging capable of exerting antioxidant and antimicrobial properties without direct contact with the food [364,365].

In addition to their antimicrobial properties, the active films derived from the conjugation of chitosan with conjugated linoleic acid (CLA) demonstrate antioxidant properties as well, thereby providing multiple protection to packaged products [366]. The electrospun nanofibers incorporated with epoxidized LA derivatives provide sustained release profiles and are particularly suitable for foods that contain a high level of moisture, which are more susceptible to microbiological degradation [364]. Additionally, films made from polylactic acid (PLA) containing 13-HODE are effective at reducing oxygen permeability and preventing microbiological spoilage of fruit and vegetables [367,368].

#### 5.2.6. Regulatory and Sensory Considerations

Despite the fact that many linoleic acid derivatives, such as conjugated linoleic acid (CLA), are generally considered safe (GRAS), some oxidized forms may need further toxicological evaluation, particularly when used at concentrations greater than normal physiological levels. The importance of this requirement is highlighted in the context of their application in functional foods or active packaging, where exposure is likely to be continuous and cumulative [369,370].

In the absence of appropriate encapsulation and masking strategies, certain epoxidized and oxo-derivatives of LA may impart metallic, grassy, or rancid notes to foods. While these compounds are bioactive, if they are not formulated properly, they can compromise the organoleptic acceptability of the final product [371].

Controlling the migration of active compounds into the food matrix is critical for consumer safety in packaging applications. Hence, it is imperative that the limit values imposed by competent authorities, such as the EFSA and the FDA, be strictly observed. In general, migration is not permitted to exceed 10 mg/dm^2^ of food contact surface area [372,373].

It is essential to increase transparency in the communication of the functions and the origin of bioactive ingredients as part of clean label initiatives. In accordance with consumer information requirements and the current trends in the food industry toward sustainability and naturalness, the LA derivatives used as natural antioxidants or antimicrobials should be clearly labeled as “antioxidants of natural origin” or “antimicrobial agents of plant origin” [374].

### 5.3. Potential as Flavor Enhancers and Nutritional Fortifiers

As an emerging multifunctional ingredient in the matrix of functional foods and beverages, linoleic acid (LA) and its ecologically synthesized derivatives, such as hydroxy-, epoxy-, oxo-, and conjugated versions, stand out [344,375]. Furthermore, these derivatives also possess dual functions, contributing to both improving the sensory profile (taste, smell, texture) and optimizing nutritional intake in addition to their primary role as bioactive agents with antioxidant, anti-inflammatory, and antimicrobial properties [376,377].

Recent developments in the field of applied lipidomics and volatile compound chemistry have revealed a complex network of interactions between lipid metabolites and sensory perception systems, including taste and olfactory receptors, as well as neuroendocrine signaling pathways regulating appetite and satiety [378,379,380]. Molecular interactions may not only play a significant role in determining the hedonic attributes of food, but can also influence systemic physiological responses via functional axes such as gut–brain or gut–phosphate [381,382,383].

In light of this, linoleic acid derivatives can be considered a new class of bioactive food constituents with high added value that can assist in the development of functional food products of the next generation. An advanced nutrition concept based on personalization and sustainability integrates therapeutic efficacy and sensory acceptability in a way that is synergistically effective.

#### 5.3.1. Flavor Modulation Through Lipid-Derived Volatiles

In foods subject to thermal processing, fermentation, or high fat content, linoleic acid (LA) and its oxidized derivatives play a key role in the generation of aromatically active volatile compounds [371,384,385]. The oxidation of linoleic acid by enzymes and non-enzymes generates a variety of aldehydes, ketones, alcohols, and lactones, which contribute to the specific flavor profile of various foods [384,386]. Depending on the concentration, matrix, and technological context of the product, these volatile compounds can have a significant effect on sensory perception [387].

Green synthesis derivatives, such as 13-hydroxy-octadecadienoic acid (13-HODE) and 13-oxo-octadecadienoic acid (13-KODE), can be further processed into volatile molecules responsible for characteristic aromatic notes [388]. Compounds such as hexanal, 2-pentylfuran, and trans-2-nonenal, for example, impart vegetable, nutty, or fatty notes and are commonly found in cereals and legumes [389,390,391]. Derived from hydroxy derivatives of LA, γ- and δ-lactones contribute to the development of sweet, creamy, coconut-like flavors specific to the dairy matrix and confectionery [392,393]. For example, fermented dairy beverages fortified with 13-HODE, which had been produced in situ, demonstrated increased concentrations of flavor compounds, including 1-octen-3-ol and 2-nonenal, that were positively correlated with sensory acceptability scores obtained in test panels [394,395].

Additionally, linoleic acid derivatives may indirectly contribute to enhanced umami perception and improved oral texture, apart from their direct role in flavor generation. In food processing or during digestion, they may modulate the release of nucleotides and glutamates, thereby increasing the complexity of taste [371,396]. In herbal formulations, and especially in low-fat products, their lipophilic properties have a positive influence on mouthfeel and creaminess. CLA–protein complexes contribute to increased viscosity and richness, enhancing perceived flavor intensity and sensory satisfaction [397,398].

LA derivatives may exert some of these effects by interacting with G-protein-coupled receptors (GPCRs) involved in fat taste perception, such as GPR120 and FFAR1. Considering this mechanism, LA derivatives have a potential role in modulating the perception of lipid-associated flavors, opening new perspectives on their functional and sensory applications in modern foods [399].

#### 5.3.2. Interaction with the Maillard Reaction and Thermal Flavor Chemistry

Linoleic acid (LA) and its derivatives participate in a number of complex thermal reactions, including the Maillard reaction and lipid–protein interactions, which are the key processes in the development of the flavor profile of baked, fried, or heat-treated foods. In highly heat-processed foods, these reactions lead to the production of volatile compounds that have a significant sensory impact and are essential to the production of the roasted, nutty, or umami notes that are characteristic of these foods [371,400].

During the Maillard reaction, oxidized derivatives of LA provide functional carbonyl groups that can react with free amino acids or peptides to form volatile heterocyclic compounds such as pyrazines and thiazoles. Compounds of this type are associated with the aroma of roasted, nutty, or complex flavors [401]. At elevated temperatures, epoxidized derivatives of linoleic acid can undergo epoxide ring-opening reactions, generating reactive aldehydes and oxygenated alkenes (alkenali), which contribute to the complexity of aromatic compounds [402,403].

Consequently, linoleic acid derivatives play a crucial role in optimizing the sensory character of thermo-processed foods, as well as in contributing to their oxidative stability and nutritional value. Due to their chemical–functional versatility, these ingredients are widely used in the development of modern functional foods with a sophisticated flavor profile and high added value.

#### 5.3.3. Nutritional Fortification: Enhancing Functional Lipid Profiles

In the context of nutrition, linoleic acid (LA) and its ecologically synthesized derivatives offer multiple opportunities for fortifying foods and beverages with bioactive lipids that support cardiometabolic, immune, and cognitive health [404,405,406]. As essential fatty acids and biochemical modulators of complex physiological pathways, these compounds are valuable ingredients for customized nutritional formulations.

Linoleic acid is an essential fatty acid required for the biosynthesis of arachidonic acid and eicosanoid mediators, but modern diets tend to have imbalances in the ω-6/ω-3 ratio, which may favor chronic inflammatory processes [407,408]. Structured lipids with optimized LA content can be used to fortify foods in a precision manner, correcting nutritional deficiencies without stimulating proinflammatory eicosanoid pathways [409]. The use of advanced delivery systems, such as solid lipid nanoparticles (SLNs) and nanostructured lipid carriers (NLCs), allows for controlled release and efficient absorption of LA, thus improving the status of essential fatty acids [410,411]. Moreover, formulations containing synergistic omega-3 fatty acids, such as EPA and DHA, allow for balanced supplementation, which is relevant in infant formula or geriatric nutrition products [412,413].

Several functional derivatives of LA have been shown to modulate lipid metabolism, insulin sensitivity, and inflammation, which are essential for nutritional interventions for certain pathologies. CLA isomers, including c9,t11 and t10,c12, have been associated with reduced fat mass, improved insulin signaling, and decreased levels of proinflammatory cytokines [414]. Further, 13-HODE and 9-HODE are endogenous ligands for the PPARγ receptor, influencing adipocyte differentiation and glucose homeostasis [415]. Structured lipids (SLs) containing conjugated linoleic acid (CLA) and butyric acid have been investigated for their applications in low-calorie and weight loss foods. Several methods of synthesis were evaluated, and CLA ethyl ester (CLAee) transesterification with tributyltin under vacuum was selected as an efficient method of synthesis. These CLA and butyric acid-enriched SLs have the potential to replace natural oils in food products, offering an innovative solution for obesity prevention and significant commercial potential [409].

In addition, LA-derived oxylipins, particularly epoxy- and hydroxy-oxylipins, have been examined for their neuromodulatory and neuroprotective properties. These metabolites are capable of crossing the blood–brain barrier and modulating pathways involved in the pathogenesis of neurodegenerative disorders [416,417]. The neuroprotective effects of compounds such as 9,10-EpOME and 12,13-EpOME have been demonstrated in experimental models of ischemic brain injury. By incorporating these derivatives into the matrix of functional products, cognitive function may be enhanced, particularly in elderly populations or individuals at an increased risk of neurological decline [418,419]. Therefore, linoleic acid and its derivatives obtained through sustainable technologies not only offer bioactive value, but also constitute functional tools for the development of precision nutrition, which emphasizes prevention and health optimization both at the population and individual levels.

Biosynthesized linoleic acid and its derivatives have remarkable multifunctional properties that act simultaneously as flavor profile modulators and active nutritional fortifiers. Their functionality is manifested both by contributing to the enhancement of sensory characteristics—through the generation of lipid-derived volatile compounds and by influencing the perception of oral creaminess and fullness—and by optimizing nutritional value, through the targeted delivery of bioactive lipids and the regulation of metabolic pathways involved in lipid, inflammatory, and energy homeostasis [85,371].

**Figure 7:**
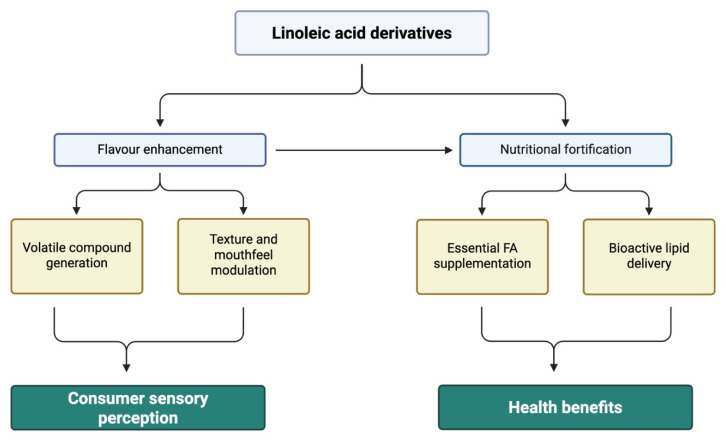
Graphical model: multifunctional role of green-synthesized linoleic acid derivatives in functional foods.

Developing advanced encapsulation and formulation strategies to protect both bioactivity and organoleptic integrity is essential for maximizing the functional efficiency of these derivatives [420]. In this regard, linoleic acid and its derivatives are considered the key components for developing next-generation nutritional solutions for prevention, nutritional therapy, and personalized nutrition, underpinned by principles of sustainability and biotechnological innovation [421,422]. Figure 7 presents the multifunctional role of green-synthesized linoleic acid derivatives in functional foods.

Table 3 presents a comparative overview of various green-synthesized linoleic acid derivatives.

## 6. Regulatory Status and Market Trends

### 6.1. Regulatory Frameworks Governing Linoleic Acid and Its Derivatives

The regulatory status of linoleic acid and its derivatives varies significantly by jurisdiction, with classification and authorization determined by chemical identity, manufacturing process, intended purpose, and levels of inclusion in food [423,424,425]. It is widely acknowledged that native linoleic acid is an essential nutrient with a well-documented safety profile and is widely accepted under international regulations. However, structurally modified or oxidized derivatives, particularly those obtained through innovative green synthesis methods, must be assessed under novel food or functional ingredient legislation [426].

Bioavailability, stability, toxicokinetics, as well as possible interactions with other food or drug components, are all considered in their evaluation. In this context, regulatory authorities such as the European Food Safety Authority (EFSA) or the Food and Drug Administration (FDA) impose stringent requirements on the characterization of the compounds, traceability of technological processes used to obtain them, and demonstration of safety at the doses suggested for human consumption. The application of this approach is essential to ensure the protection of consumers, especially in the case of bioactive derivatives that exhibit significant metabolic activity and may have an effect on systemic physiological responses [427,428].

The integration of linoleic acid derivatives into functional foods or supplements requires a layered regulatory approach in accordance with national and international legislative standards, taking into account both the technological innovation involved in their synthesis and the criteria of safety, efficacy, and correct labeling.

#### 6.1.1. United States—FDA and GRAS Status

Linoleic acid is included on the list of generally recognized as safe substances (GRAS) in foods and infant formulas [429]. Among the most widely researched derivatives of linoleic acid, conjugated linoleic acid (CLA) has gained GRAS certification and is permitted to be added to a variety of foods, including dairy products, meat analogs, and beverages. A substantial body of data regarding its safety and metabolic efficacy supports this recognition [225].

However, derivatives obtained by green synthesis methods, such as enzymatic epoxidation, microbial fermentation, or controlled photooxidation, may be subject to the Food Additives Amendment if they are considered to be significantly different in structure or function from the parent compound. It is necessary to conduct a regulatory re-evaluation in such situations to determine whether these compounds may be safely integrated into the food chain [430,431].

The FDA’s New Dietary Ingredient (NDI) notification procedure applies to dietary supplements that contain structurally modified linoleic acid derivatives that were not marketed in the United States prior to 1994. As part of this process, a detailed scientific dossier must be submitted describing the composition, mode of action, safety, and bioavailability of the proposed ingredient. In order to ensure both regulatory compliance and end-consumer protection, a rigorous approach is required in the context of nutritional innovation and sustainable biotechnologies [432,433].

#### 6.1.2. European Union—EFSA and Novel Food Authorization

Linoleic acid (LA) and its conventional derivatives, such as triglycerides, are considered essential nutrients in the European Union and are permitted in both foods and food supplements. However, green-synthesized derivatives—such as 13-HODE, 13-KODE, and oxylipins—are subject to the regulations set forth in the Novel Foods Regulation (EU) 2015/2283 and considered novel ingredients from a legislative perspective, as they have had little or no history of human consumption in the European Union prior to 1997 [434].

In order to be placed on the European market, these compounds have to undergo a complex pre-market safety assessment procedure carried out by the European Food Safety Authority (EFSA), which includes detailed toxicological analyses, including genotoxicity testing, determination of the No Observed Adverse Effect Level (NOAEL), stability and estimated exposure studies, analysis of nutritional relevance, potential interactions with other nutrients, allergenicity risk assessment, and analysis of cumulative intake from all dietary sources [435].

As an example, conjugated linoleic acid (CLA) has been authorized as a novel food in the EU with clear maximum limits (up to 3 g/day for adults) and strict compositional specifications (minimum 80% active isomers and maximum 1% trans fat content) [436,437]. While HODE and green-synthesized oxylipin derivatives have not been approved for marketing in the EU, several dossiers are currently in the preliminary stages of preparation or pre-submission [438].

This rigorous approach reflects the commitment of European regulators to the protection of public health and the need for sound scientific documentation for innovative ingredients, especially in the context of the growing interest in bioactive compounds derived through sustainable and environmentally friendly technologies.

#### 6.1.3. Asia–Pacific and Codex Alimentarius Considerations

Regulations in jurisdictions such as China, Japan, and South Korea are generally aligned with those set forth by Codex Alimentarius, with specific applications depending on the product category and target population [439]. The use of linoleic acid (LA) in infant formula and fortified foods is authorized, and LA is recognized as an essential nutrient [440]. Several derivatives of linoleic acid, including conjugated linoleic acid (CLA), are acceptable under certain conditions, subject to restrictions on labeling and consumption [441].

The National Health Commission (NHC) in China has approved the use of CLA in health foods, but imposes strict restrictions on its inclusion in beverages intended for infant consumption due to safety and physiological suitability considerations [442]. The FOSHU (Foods for Specified Health Uses) regulatory framework in Japan allows the use of CLA-based formulations provided there is adequate clinical evidence to support the health claims made [375,443]. As part of this approach, rigorous scientific validation is required for the functional effects, as well as compliance with quality, purity, and dosage regulations established by the appropriate regulatory agencies.

At the international level, Codex Alimentarius has not yet published monographs or standards on oxidized linoleic acid derivatives obtained by green synthesis, indicating a gap in global regulation for new categories of functional lipid ingredients. As a result of the lack of harmonized standards, the uniform adoption of these compounds in different markets is limited. Therefore, it is essential that international regulatory frameworks be developed that incorporate both safety criteria and principles for assessing the biological efficacy and sustainability of manufacturing processes. In the context of the expansion of functional foods and the application of personalized nutrition principles on the global level, global standardization becomes increasingly important [444].

#### 6.1.4. Market Trends and Commercialization Strategies

The global market for functional lipids, in particular omega-6 derivatives such as linoleic acid (LA) and conjugated linoleic acid (CLA), is going through a phase of accelerated expansion driven by the convergence of three key factors: increasing consumer demand for ingredients of natural origin, the food industry’s shift towards transparently labeled products according to clean label principles, and the strengthening of the scientific basis validating the bioactivity of these compounds [445,446].

This trend reflects a fundamental shift in the global nutritional paradigm, where consumers no longer seek foods with only basic nutritional value, but prefer products with dual functionality—nutritional and physiological—that can support cardiometabolic, immune, or cognitive health [447,448]. As a result of developments in sustainable biotechnologies and green synthesis, new lipid derivatives are being developed with improved efficacy and safety profiles, which can meet the demands of the global market for functional foods and advanced nutritional supplements [449].

In fact, omega-6 derivatives, in particular, those derived from linoleic acid, are not only essential nutritional vectors, but also catalysts of food innovation, contributing to the development of a new segment of products that are dedicated to personalized, sustainable, and science-based nutrition [450].

#### 6.1.5. Global Market Dynamics

The conjugated linoleic acid (CLA) market is expected to reach USD 230 million in 2023 and USD 422 million by 2030, corresponding to an average compound annual growth rate (CAGR) of 5.4% [451]. This growth momentum is primarily driven by the increasing prevalence of metabolic syndrome and obesity, the increasing integration of CLA into supplements for sports nutrition and weight loss management, and the expanding food segments including dairy products and plant-based alternatives fortified with CLA [280,452].

In addition to the scientific validation of CLA’s beneficial effects on lipid metabolism and systemic inflammation, consumers prefer functional ingredients with a natural profile and clinical evidence [453]. In parallel, other green-synthesized linoleic acid derivatives, such as structured lipids, 13-HODE, and oxylipins, are in the early stages of innovation and development and are being developed for integration into emerging market segments, particularly personalized nutrition and clinical nutrition [454].

It is anticipated that these bioactive compounds will substantially contribute to the diversification of the functional lipid ingredient portfolio through their applicability in advanced formulations tailored to individual metabolic profiles, specific therapeutic requirements, and global trends towards sustainability, efficacy, and nutritional transparency [449].

#### 6.1.6. Key Players and Technological Differentiators

The major companies involved in the commercialization of linoleic acid (LA) derivatives are the leading international players in the field of functional ingredients, which invest strategically in the development and validation of bioactive lipid compounds. There is a number of companies in this category, including BASF SE and Stepan Lipid Nutrition, which manufacture ingredients based on CLA marketed under the brand names Tonalin^®^ and Clarinol^®^, which have been widely used in weight management products and metabolic health products [219,455]. DSM Nutritional Products is also exploring applications of enzyme-modified lipids to support brain and metabolic health, and companies such as Kancor Ingredients and Cargill are actively developing technology platforms for lipid modification utilizing green chemistry methods [454].

In this market segment, competitive advantages include green certification of synthetic processes as well as lifecycle assessments (LCAs) that quantify the sustainability of the entire production chain. Moreover, innovations in microencapsulation and advanced delivery systems, such as liposomes, cyclodextrins, and nanostructured lipid carriers (NLCs), are essential to ensure their stability, bioavailability, and functionality in food matrices [456,457].

Another key component of competitive differentiation is the generation of robust clinical data and the validation of health claims through controlled studies, which enable regulatory approval and the transparent communication of nutritional benefits to consumers and health professionals. It is intended that these strategic directions contribute to strengthening LA derivatives’ position as next-generation functional ingredients that are tailored to global market demands for efficacy, safety, and sustainability [31,404,423].

## 7. Challenges, Limitations, and Future Prospects

### 7.1. Current Limitations in Synthesis, Characterization, and Application

Despite the promising potential shown by linoleic acid (LA) and its derivatives obtained by green synthesis in applications such as functional foods, nutraceuticals, and health-oriented formulations, a number of technological and regulatory hurdles persist, limiting the industrial-scale implementation of these compounds [458]. In the context of complex food matrices, these constraints are mainly related to scalability, analytical traceability, and formulation performance [459].

These limitations arise due to the inherent complexity of lipid oxidation chemistry, which produces unstable and difficult-to-control derivatives under industrial conditions [460,461]. Interactions of LA derivatives with components of the food matrix—such as proteins, polysaccharides, or minerals—may negatively influence physicochemical stability, bioavailability, and bioactive efficacy, requiring complex optimizations of delivery systems [462,463].

Additionally, there are high expectations imposed by the international regulatory framework for the introduction of new-generation bioactive compounds on the market [1,464]. In addition, there are requirements for detailed compound characterization, validation of functionality through clinical data, demonstration of safety, and compliance with the existing food legislation. Therefore, for ecologically synthesized derivatives of linoleic acid to reach their full application potential, an integrated approach combining innovation in synthesis, advances in analytical methods for characterization, and functional validation through standardized scientific protocols is required [464,465].

#### 7.1.1. Challenges in Green Synthesis Pathways

The industrial-scale implementation of linoleic acid (LA) and derivatives derived from green synthesis has been limited by a number of technological and regulatory bottlenecks, despite the promising potential shown by LA and its derivatives, including functional foods, nutraceuticals, and health-oriented formulations. These constraints mainly concern the scalability of synthesis, analytical traceability, and formulation performance in the context of complex food matrices [84,197,466].

Due to the inherent complexity of lipid oxidation chemistry, these limitations result from the multistep formation of unstable and difficult-to-control derivatives under industrial conditions [460,467]. Moreover, the strict specificity of the enzymes involved in the green synthesis of these compounds limits the yield and flexibility of the process, especially in the context of stereoselective conversions or microbially mediated reactions [468,469,470]. The interaction between LA derivatives and food matrix components—such as proteins, polysaccharides, or minerals—may negatively affect their physicochemical stability, bioavailability, and bioactive efficacy, requiring complex formulations of delivery systems [462].

Additionally, the international regulatory framework imposes high requirements for placing new-generation bioactive compounds on the market. The requirements include detailed compound characterization, clinical validation of functionality, demonstration of safety, and compliance with the existing food legislation. Therefore, for ecologically synthesized derivatives of linoleic acid to reach their full application potential, an integrated approach combining innovation in synthesis, advances in analytical methods for characterization, and functional validation through standardized scientific protocols is essential [464].

#### 7.1.2. Analytical and Characterization Limitations

Analyzing linoleic acid (LA) derivatives in complex food matrices can be challenging due to the complexity of their identification, accurate quantification, and structural elucidation. There are several reasons for these difficulties, including the chemical lability of these compounds, the low concentrations at which they are found in final products, and the wide range of isomeric structures within their molecules [471].

Derivatives obtained by green synthesis, such as HODEs and oxo- and epoxy-derivative acids, are often formed as mixtures of regio- and stereoisomers, which considerably complicates the structural assignment process [472]. Conventional chromatographic methods, such as GC–MS or LC–UV, do not provide sufficient resolution to differentiate between positional isomers (e.g., 9-HODE vs. 13-HODE), and advanced chiral chromatographic techniques or two-dimensional (2D) NMR spectroscopy, although capable of enantiospecific characterization, involve high costs and extended analysis time [473,474]. The use of tandem mass spectrometry (MS/MS), although widespread, can generate in-source oxidation artifacts, leading to false-positive results in complex lipidomic analyses [475,476].

A low recovery rate and reduced extraction efficiency can also be caused by interactions between oxidized lipid compounds and other components of the food matrix, such as proteins, polysaccharides, or metal ions [477,478]. Sample preparation techniques, such as solid-phase extraction (SPE) or QuEChERS-type protocols, require individualized optimization for each type of matrix, which limits transferability and methodological standardization [479,480,481]. In addition, the lack of accessible or economically viable isotopically labeled internal standards is an additional barrier in the development of robust validated methods [482].

An important problem is the absence of harmonized analytical standards, particularly for new linoleic acid derivatives such as oxylipins or structured lipids. There is a lack of such references, which hinders the comparability of health claims between laboratories, regulatory compliance, and the validation of health claims. In the context of growing interest in green-synthesized oxidized compounds, where analytical rigor is essential for commercial and regulatory acceptance, this shortcoming is of particular importance. The development and standardization of integrated analytical platforms capable of addressing both isomeric complexity and matrix interferences is a fundamental prerequisite for advancing the industrial applications of these bioactive compounds [483,484].

#### 7.1.3. Formulation and Application Constraints

Although green-synthesized linoleic acid (LA) derivatives have considerable biological and technological benefits, their use in functional food formulations remains restricted because of limitations related to physicochemical instability, negative sensory impact, and inefficiency of conventional delivery systems. Consumer acceptability and biological efficacy of activated compounds are both affected by these constraints [467,485,486].

One of the most significant challenges is the oxidative instability of these compounds, particularly in the case of epoxy-derivatives and hydroxy-fatty acids. They are susceptible to autoxidation, isomerization, and hydrolysis under typical food processing and storage conditions [487,488]. Parameters such as pH, temperature, and exposure to oxygen cause accelerated degradation of the compounds, significantly reducing the shelf life of the product [477]. In many cases, conventional antioxidant co-formulants, such as tocopherols or rosemary extracts, do not provide sufficient long-term protection, requiring the development of advanced stabilization strategies [460,487,489].

Sensory characteristics of some LA derivatives may be undesirable, especially when incorporated in functionally relevant concentrations, despite their recognized nutritional value. A large number of epoxy- and oxo-derivatives exhibit increased reactivity towards volatile compounds with amine groups, which may significantly alter the final product’s flavor profile [371,490,491]. Although encapsulation or masking strategies, such as the formation of cyclodextrin complexes, can reduce the sensory impact, their applicability is often limited by high costs, and they are difficult to implement in products intended for mass consumption [492].

The bioavailability of these compounds, coupled with their susceptibility to gastrointestinal degradation, requires sophisticated delivery systems such as liposomes, nanoemulsions, or nanostructured lipid carriers [493,494]. However, the limited scalability and high production costs of these technological vehicles restrict their widespread adoption in the food industry. In addition, the lack of in vivo pharmacokinetic data for most green-synthesized derivatives limits formulation optimization and delays clinical validation of functional benefits, thus affecting the transition from the laboratory to the market [495,496,497].

As a result, the efficient valorization of LA derivatives in functional foods requires overcoming these challenges by integrating multidisciplinary solutions that ensure stability, efficacy, and acceptability within the context of industrial application.

### 7.2. Scale-Up Challenges for Industrial Production

Transforming green synthesis protocols of linoleic acid (LA) derivatives from the experimental laboratory to robust, reproducible, and economically sustainable industrial processes involves major engineering, bioprocessing, and regulatory challenges. Due to the interplay between the unique physicochemical and biological characteristics of LA derivatives, the need to adhere to strict environmental principles when processing, and the regulatory restrictions imposed on bioactive ingredients for food or nutraceutical use, these challenges are highly complex [498,499].

Derivatives obtained by green synthesis show sensitivity to such factors as temperature, pH, oxygen, and humidity, which requires the use of mild and controlled reaction conditions [384,500]. As well as requiring high-purity substrates, precisely buffered systems, and fine optimization of reaction times, many enzymes or microorganisms involved in the catalytic transformations of LA significantly complicate the design and operation of industrial-scale processes. The maintenance of these conditions can be a challenge in high-volume production units, where scaling factors can negatively affect catalytic efficiency and the overall yield [501].

The feasibility of using enzymatic, microbial, and chemocatalytic pathways for the synthesis of LA derivatives has been demonstrated through proof-of-concept studies; however, the scale-up process remains a major barrier to the market penetration and commercialization of these compounds. The intermediate stabilization, purification, and formulation steps, as well as the need for toxicological and nutritional evaluation, further complicate the production process. Additionally, regulatory requirements related to safety, traceability, labeling, and quality impose rigorous validation, auditing, and compliance procedures, requiring considerable resources and a multidisciplinary approach [84].

Therefore, the conversion of green synthesis processes from the laboratory to scalable industrial applications cannot be handled in isolation, as it requires a coherent integration between process engineering, biocatalysis, the development of eco-friendly technologies, and a comprehensive understanding of the regulatory framework. An integrated approach is the only means by which linoleic acid derivatives can realize their full potential in the context of sustainable functional foods and precision nutrition.

#### 7.2.1. Process Engineering Constraints in Enzymatic Synthesis

In order to obtain bioactive compounds such as HODEs, EpOMEs, and structured lipids, enzymes such as lipoxygenases, cytochromes P450, or epoxygenases can be used in the enzymatic synthesis of linoleic acid (LA) derivatives. There are, however, a number of major technological challenges involved in translating these reactions from laboratory scale to industrial scale, including catalyst stability, reaction kinetics, and bioreactor design [502,503].

There is a critical hurdle associated with the use of free enzyme systems, as they are fragile and costly when used repeatedly. Increasing operational stability and reducing costs in large-scale synthetic processes is achieved by immobilizing enzymes on solid supports, such as silica, chitosan, or metal–organic frameworks (MOFs) [504,505,506]. Nevertheless, this approach is limited by difficulties in maintaining catalytic activity, specificity, and reusability of immobilized enzymes, especially in conditions of high industrial flux. Furthermore, such systems often suffer from mass transfer limitations and non-uniform enzyme distribution in fixed-bed or fluidized-bed reactors, reducing their overall catalytic efficiency [507].

LA’s enzymatic transformations are often limited by substrate availability, and reaction behavior deviates from the classical Michaelis–Menten model, especially at higher concentrations where substrate or product inhibition occurs [508]. As far as reactor design is concerned, stirred-tank batch bioreactors are not suitable for enzymatic reactions involving LA due to problems with oxygen transfer, heat dissipation, and interfacial enzyme deactivation. To overcome these limitations, advanced bioreactor configurations such as membrane bioreactors, segmented flow microreactors, or tubular plug flow systems are required. These technologies, however, require a substantial amount of investment and high operational complexity, which may limit their adoption by small and medium-sized enterprises (SMEs), which have limited technological and financial resources [509,510].

In conclusion, optimizing the scale-up of the enzymatic synthesis of linoleic acid derivatives requires an integrated approach encompassing the development of sustainable biocatalysts, advanced modeling of reaction kinetics, as well as engineering solutions tailored to the efficiency, yield, and sustainability requirements of industrial production.

#### 7.2.2. Bottlenecks in Microbial Fermentation Processes

Genetically modified bacteria, fungi, or yeast strains have been used to produce linoleic acid derivatives in recent years due to their potential to provide high reaction specificity and the ability to utilize renewable feedstocks. Despite this, industrial applications of these bioprocesses face complex challenges related to robustness, scalability, and operational sustainability [511,512,513].

A major concern is the robustness and productivity of engineered microbial strains. As a result of genetic modifications, metabolic stress is often introduced, resulting in reduced growth rate, genetic instability, and increased mutation rates during long fermentations. Ensuring strain phenotypic and genotypic stability across multiple passages and in large fermentation volumes (≥1000 L) is essential, but rarely reproducibly demonstrated. Efforts are being made to optimize process conditions to increase volumetric yields (g/L/h), but current yields are below the threshold for economic viability for commercial applications [514,515,516].

Another limiting factor is the operational conditions in bioreactors. As a result of microbially expressed enzymes catalyzing linoleic acid hydroxylation and epoxidation, increased oxygen supplies are required, which necessitates the use of bioreactors with intensive aeration [517,518]. As a result, excessive foaming occurs, and high energy consumption is required, which reduces the efficiency of the process and increases the environmental impact. Furthermore, the recovery of products from the fermentation medium is challenging, as these compounds are often emulsified, embedded in cell membranes, or bound to intracellular fractions. It is common to perform purification steps using organic solvents, centrifugation, or advanced technologies such as supercritical CO_2_ extraction, which present additional challenges in terms of sustainability and operational costs [519].

Another critical issue is the variability of the feedstocks that are used as fermentation substrates. On an industrial scale, sources such as glucose, glycerol, or waste lipids are commonly used. The fatty acid composition of these sources may differ, as well as the contaminant content (e.g., waste oils or crude microbial oils), which can affect batch-to-batch consistency and quality of the final product [520,521].

To overcome these limitations, advanced metabolic engineering, bioprocessing, and green purification strategies, as well as standardization of raw materials and optimization of logistics, are required. The commercial feasibility of microbial biosynthesis of linoleic acid derivatives in a circular economy can only be achieved through this systemic approach [522,523].

#### 7.2.3. Challenges in Photocatalytic and Electrochemical Oxidation

In spite of the significant advantages provided by photochemical and electrochemical methods for producing linoleic acid (LA) derivatives, including increased atomic efficiency and environmental compatibility, it is difficult to translate these processes to industrial scale due to the complexity of reactor architecture, the control of reaction, light distribution, and electrode material selection [524,525].

Light penetration is one of the key limiting factors in photocatalytic oxidation reactions. It is difficult to maintain uniform irradiance in systems with high turbidity, such as feed emulsions or oils with high viscosities. A critical role is played by reactor design in this context. Despite the improved surface-to-volume ratio offered by flat-panel or tubular configurations, advanced engineering solutions are required to ensure a constant laminar flow and uniformity of photon flux throughout the reactor section [526,527]. Heterogeneity in light distribution reduces the conversion efficiency and leads to the formation of byproducts or structural inhomogeneities in the resulting compounds [528].

Electrochemical processes, although attractive in terms of avoiding the use of additional chemical reagents, are plagued by problems associated with electrode fouling, gas bubble formation that interferes with the active surface, and reduced selectivity when the cell size exceeds laboratory standards. Electrodes with high performance, such as boron-doped diamond or titanium oxide electrodes, provide efficient conversions, but they are costly and are limited in supply [529,530]. To prevent overoxidation of substrates and the generation of unwanted secondary compounds, the density of the current must also be maintained within strictly controlled ranges [531].

The industrial applicability of these processes depends largely on the development of integrated solutions that combine reactor engineering, mass and energy transfer optimization, the selection of functional materials, and the implementation of high-fidelity reactive control techniques. The use of photochemical and electrochemical routes for the production of LA derivatives remains limited to experimental and pilot scales, with significant obstacles to commercialization.

#### 7.2.4. Process Integration, Purification, and Formulation

In situations where environmentally friendly methods are used to synthesize LA derivatives, subsequent steps such as purification, concentration, drying, and formulation can introduce additional challenges in industrial scale-up. Although these steps are essential to obtaining a stable, standardized end product capable of meeting regulatory and commercial requirements, they are associated with high operational costs and a great deal of technological complexity [532,533].

The first major hurdle is the purification of isomeric mixtures resulting from green synthesis. LA derivatives, such as HODEs, oxylipins, or epoxy-fatty acids, are frequently generated as mixtures of regio- and stereoisomers, which require high-performance chromatographic separation or molecular distillation [534,535]. There are significant resource requirements associated with these methods, and they are difficult to scale up from laboratories to industrial scale without compromising the efficiency or purity of the process. Due to the lack of enantiomeric standards for these compounds, batch-to-batch standardization is further complicated, affecting the consistency of finished products [536].

There are also significant challenges associated with the drying and microencapsulation steps. Spray-drying and freeze-drying of lipid formulations, such as CLA-based microcapsules, require thermostable carriers such as maltodextrin or modified starch, as well as compatible emulsifiers. These excipients affect not only the technological stability of the product, but also regulatory issues and consumer perception, especially when it comes to clean-label labeling [537,538,539].

Furthermore, environmental parameters and packaging conditions must be strictly controlled to ensure encapsulation efficiency and oxidative stability of the compounds. It is possible for oxidative degradation or migration losses to significantly reduce the bioactivity of the product and compromise its functional efficacy [540,541,542,543].

Consequently, post-synthesis steps are critical to determining the commercial viability of LA derivatives generated by green synthesis, requiring both functional performance and regulatory compliance of the final product, which requires integrated technological solutions and multidisciplinary optimizations.

### 7.3. Future Directions for Research and Development

Research and development (R&D) in the field of linoleic acid (LA) and its structurally modified derivatives is at an inflection point as the intersection of green chemistry, lipid biotechnology, and functional food science continues to develop. Although substantial progress has been made in identifying the bioactivity of these compounds and developing sustainable synthetic strategies, a number of unexplored directions remain for precision molecular customization, targeted delivery, and clinical translation of functional lipid compounds [544,545].

The value of these derivatives lies not only in their fundamental biological functions, but also in their ability to be integrated into the food matrix through advanced technological designs tailored to the individual metabolic profile and precision nutrition requirements [546]. Therefore, future innovations will be highly dependent on the integration of several converging disciplines, such as synthetic biology (for optimizing biosynthetic pathways), materials science (for developing intelligent delivery and encapsulation systems), nutritional metabolomics (for decoding personalized metabolic effects), and advanced food processing technologies (for ensuring the finished product is stable, functional, and acceptable) [547,548,549,550].

This transdisciplinary approach will not only enhance the physicochemical properties of LA derivatives, but also expand their application in frontier areas such as clinical nutrition, personalized dietary therapies, or next-generation functional foods. For this reason, it will be necessary to build an effective and scalable nutritional tool from these bioactive lipids through collaboration between academic research, industry, and regulatory bodies in order to ensure a coherent, sustainable knowledge transfer that has a significant social impact.

#### 7.3.1. Advanced Green Synthesis Pathways and Biotechnological Innovations

A key strategic R&D frontier in the field of linoleic acid (LA) derivatives involves the rational design of microbial “cell factories” for the personalized biosynthesis of these compounds with high stereochemical precision and high industrial yields [551,552]. Recent advances in CRISPR-based genome editing, modular metabolic pathway design, and dynamic regulation of genetic circuitry have made it possible to construct next-generation chassis organisms capable of producing specific LA derivatives on an industrial scale, including *Yarrowia lipolytica*, *Corynebacterium glutamicum*, and *Rhodosporidium toruloides* [553,554,555].

It is possible to program these optimized microbial systems to synthesize compounds of significant interest, including 13-HODE and 9-HODE with defined stereochemistry, epoxy-linoleates with 12,13-EpOME, well-known for their neuroprotective and vasculotropic properties, as well as hydroxy- and lactonized derivatives with improved solubility and bioactivity [556,557,558]. With the implementation of dynamic metabolic control strategies, such as sensor-regulatory loops with automatic feedback, the desired metabolic fluxes can be maintained and byproducts avoided, resulting in the optimization of three critical performance parameters—titer, rate, and yield—collectively referred to as TRY [559,560].

The identification and optimization of enzymes capable of catalyzing highly specific modifications at the level of the LA molecule is another fundamental pillar for advancing the biosynthesis of LA derivatives. Researchers should explore microbial biodiversity to discover lipoxygenases, epoxygenases, and hydroxylases that have precise spatial and regioselective specificities and then engineer these biocatalysts using directed evolution technologies supported by high-throughput screening platforms [561,562].

For the purpose of active site remodeling, modern enzyme engineering approaches use computational docking algorithms and structure-assisted design techniques [563]. Fusion proteins, which integrate biocatalysts into multifunctional enzyme chains, can also facilitate cascade reactions within the same reactor, thereby reducing process times and minimizing intermediate losses [564,565,566].

Using these cutting-edge biotechnological solutions, oxidized, epoxidized, and conjugated linoleic acid derivatives will be synthesized precisely and efficiently under mild, aqueous, and green chemistry-compatible conditions. As a result of this streamlined biosynthesis paradigm, next-generation functional ingredients can be developed in a sustainable manner, providing scalable platforms that can be applied in personalized nutrition, metabolic health, and precision dietary therapy.

#### 7.3.2. Integration of Green Chemistry with Smart Formulation Technologies

It is essential to develop innovative delivery systems that overcome the limitations associated with chemical instability, low solubility, and low bioavailability when using eco-synthesized linoleic acid (LA) derivatives in functional foods and nutraceuticals. For these bioactive compounds to be effectively incorporated into the food matrix, multifunctional platforms must be developed that provide protection, controlled release, and targeted absorption in the gastrointestinal tract or target tissues [287,561,567].

Current research should focus on the development of next-generation encapsulation systems incorporating nanoparticles sensitive to physiological stimuli (pH, temperature, enzyme activity), which will release LA derivatives according to the local conditions of the biological environment in a controlled manner [568,569,570,571]. Furthermore, coacervate-based multilamellar microcapsules can deliver these derivatives alongside other bioactive compounds that promote metabolic and functional efficiency, such as polyphenols [572,573,574].

Protein–lipid hybrid micelles are another promising concept that can selectively interact with intestinal epithelial transporters, such as CD36 and FATP4, to enhance active and targeted absorption of bioactive lipids. In addition to protecting the LA derivatives during technological processing and storage, these advanced systems should maintain their bioactivity until they are released into the targeted physiological site [575,576].

A new research direction aims to understand and capitalize on self-assembly mechanisms in complex matrices by studying the interfacial interactions between LA derivatives and food biopolymers. Food proteins, such as caseins and legumes, can interact with LA derivatives and influence their physicochemical stability and emulsifying properties [577,578]. Furthermore, the interaction of lipids with polysaccharides (e.g., pectin or carrageenan) may improve the rheology and functional stability of formulated products [579,580,581].

Additionally, co-delivery strategies of LA derivatives with prebiotics or probiotic strains offer new perspectives for synergistic nutritional interventions targeting gut health. The combination of these compounds may improve not only absorption and biological efficiency, but also favorable microbiota remodeling, amplifying the immunometabolic effects of bioactive lipid compounds [582,583,584].

The future of nutraceutical applications of LA derivatives is, therefore, dependent on developing innovative, multifunctional, and environmentally sustainable delivery systems utilizing interdisciplinary principles combining materials science, food engineering, cell biology, and metabolomics.

#### 7.3.3. Expanding the Biological Understanding and Therapeutic Potential

Even though the existing studies have demonstrated the anti-inflammatory, antioxidant, and metabolic benefits associated with linoleic acid (LA) derivatives, more research is needed to understand their specific molecular mechanisms, tissue-specific effects, and long-term outcomes [585,586,587]. A mechanistic approach is essential in order to move from functional generalizations to personalized nutritional advice.

To gain a comprehensive understanding of how LA derivatives are metabolized, transported, and interact with cellular regulatory systems, future investigations should utilize advanced multi-omics platforms [588,589]. Lipidomic and oxylipin profiling will enable mapping of metabolic fluxes and inter-derivative conversions, while transcriptomics and epigenomics will facilitate the understanding of interactions with the key nuclear receptors involved in the homeostasis of lipids, inflammation, and energy metabolism [417,590]. It is anticipated that the emerging spatial metabolomics technologies and single-cell analyses will enable the identification of specific effects in target tissues—adipose, liver, gut, and neural—revealing the heterogeneity of the biological response at the cellular level [591,592].

Physiologically relevant in vitro models such as dynamically perfused intestinal systems and human 3D organoids can be used to test exposure to LA derivatives under controlled conditions, and humanized animal models can provide essential information for preclinical translation to human applications [593,594].

To validate the effectiveness of these compounds, longitudinal interventional clinical trials are required that investigate the safety, pharmacokinetics, and health effects of LA derivatives (encapsulated or advanced formulated) in targeted cohorts. Among the target groups are individuals with metabolic syndrome, cardiovascular disease risk, and inflammatory bowel disease, for whom effects on lipid profile, insulin sensitivity, and inflammatory markers are being investigated [404,595]. Those at risk of neurodegenerative disease or those with a history of dementia may also benefit from the neuroprotective effects of oxylipins, as well as active populations or athletes who may benefit from derivatives offering anti-inflammatory properties and a role in maintaining lean body mass [596].

It is anticipated that the integration of nutrigenomics, gut microbiome sequencing, and digital health monitoring tools (wearables, applications) will allow the stratification of individual responses, contributing to the foundation of a precision lipid nutrition paradigm [597,598,599] In this framework, linoleic acid derivatives will be formulated and recommended in a personalized manner according to the genetic, microbiotic, and metabolic profile of each individual, opening opportunities for nutritional interventions with increased efficiency and demonstrable clinical relevance in the future.

#### 7.3.4. Regulatory Science and Harmonization

Including green-synthesized linoleic acid (LA) derivatives into food and nutraceuticals in a safe, efficient, and regulated way requires future R&D activities to work closely with regulatory science and circular economy initiatives. This convergence is essential to facilitate regulatory acceptance, support process sustainability, and promote responsible innovation [600].

As a priority objective, it is essential to standardize the analytical methods used for the identification, quantification, and isomer characterization of LA derivatives. To ensure comparability of data and substantiation of regulatory dossiers, certified reference materials (CRMs) and inter-laboratory harmonization of analytical protocols should be developed [601]. Inclusion of these derivatives in official monographs, such as those published by the United States Pharmacopoeia (USP) or in EFSA scientific dossiers, will strengthen their position in the portfolio of food and functional ingredients with a clear regulatory status [602,603].

Parallel to this, harmonized frameworks are required for the assessment of the safety of oxidized lipids, including specific parameters regarding electrophilic activity, genotoxic potential, and permissible exposure levels. By using this approach, predictive risk control and transparent integration of LA derivatives into the food chain will be possible [604,605,606].

As part of the design and optimization of the green synthesis of LA derivatives, techno-economic analysis (TEA) and life cycle assessment (LCA) tools should be applied from the beginning to quantify the environmental impact and sustainability benefits associated with the processes used [607,608]. In such a holistic approach, critical points in the production chain can be identified and optimized according to environmental, economic, and social factors [609,610].

Using lipid residues from agro-industrial sources, such as oilcakes from seed pressing or microbial oils from biorefineries that valorize wastewater, is a valuable source of renewable feedstock for the environmentally friendly synthesis of LA derivatives [611,612,613]. Furthermore, the use of process co-products, such as glycerol and short-chain fatty acids, may enable the development of circular lipid-based biorefineries, thereby optimizing resource use and reducing waste [614,615].

The development of a sustainable trajectory for the large-scale integration of linoleic acid derivatives into future food systems is being achieved by aligning R&D strategies with regulatory requirements and the principles of circular bioeconomy, thereby simultaneously contributing to consumer safety, environmental protection, and responsible innovation [616].

The future of the development of linoleic acid (LA) and its ecologically synthesized derivatives is fundamentally dependent on the convergence of four key directions: precision biosynthesis, smart formulation, mechanistic validation, and regulatory integration [236]. These compounds, at the interface of molecular biology, food technology, and translational nutrition science, are poised to evolve from niche bioactive ingredients to the key components of next-generation functional foods, therapeutic nutrition, and sustainable health platforms [85,617,618].

Precision biosynthesis, supported by advanced metabolic engineering and optimized cellular platforms, will enable the controlled production of derivatives with defined stereochemistry and specific functional properties. In parallel, the development of “smart” formulations capable of providing protection, targeted release, and efficient absorption will maximize biological efficiency in diverse nutritional contexts [561]. Validation of the molecular mechanisms involved in the interactions of these compounds with human metabolic systems through multi-omics technologies and relevant preclinical models will support the scientific substantiation of functional claims. Integrating these findings into the international regulatory framework through harmonized standards, rigorous safety assessments, and transparent labeling strategies will facilitate the widespread acceptance and use of these biofunctional lipids [464,619,620].

Developing these directions will require close trans-disciplinary collaboration between academic researchers, industrial partners, and regulators. Only through a concerted cross-sectoral effort can the full potential of linoleic acid derivatives as innovative solutions for personalized nutrition, preventive health, and food sustainability be realized. This integrative vision will enable the transition of these compounds from the experimental realm to a central role in the design of next-generation functional foods and global health-oriented nutrition technologies.

## 8. Conclusions

Linoleic acid, an omega-6 fatty acid essential to human health, plays an important role in maintaining the integrity of biological membranes and in essential cellular functions. Due to its variety of sources and nutritional importance, its derivatives, especially those produced through green synthesis, have attracted increased interest.

The development of bioactive compounds with antioxidant, anti-inflammatory, and antimicrobial properties, which are relevant to the nutraceutical industry, is made possible by sustainable production methods, such as enzyme catalysis or microbial transformations. Through the use of functional products or supplements, they can contribute to improving the health of the population and extending food shelf lives.

For an effective integration into food products, challenges related to stability, bioavailability, sensory acceptability, and regulations must be overcome. In the context of a sustainable food industry, further research and interdisciplinary collaboration are required to fully exploit the nutraceutical potential of linoleic acid derivatives.

## Figures and Tables

**Table 1:** Dietary sources of LA.

Source	Foods	Approximate Amount of LA (per 100 g)
Vegetable oils	Flaxseed	14%
	Canola	19%
	Soybean	50%
	Corn	53%
	Safflower	76%
	Palm	10%
	Peanut	33%
	Cottonseed	52%
	Sunflower	44–75%
	Olive	9%
	Linseed	6%
	Rapeseed	5%
Nuts and seeds	Hemp	34%
	Black walnuts	27%
	Chia	6%
	Brazil nuts	24%
Animal products	Lard	10–15%
	Whole milk	0.1%
	Bacon	6%
	Egg	1.1%
	Butterfat	3%
	Margarine	24%
	Chicken	1.6%
	Turkey	2.8%
	Beef	0.4%
	Lamb	0.01%
	Muscles of pigs	32%
	Adipose tissue of pigs	10%

**Table 2:** Key differences between chemical and enzymatic synthesis approaches.

Aspect	Chemical Synthesis	Enzymatic Synthesis
Selectivity	Low (isomer mixtures)	High (regioselective)
Reaction conditions	High temperature, organic solvents	Mild, aqueous, or solvent-free
Environmental impact	Generates hazardous waste	Biodegradable, eco-friendly
Scalability	Well-established, industrial-scale	Advancing with enzyme immobilization
Product purity	Requires extensive purification	High purity with minimal byproducts
Industrial application	Large-scale conventional processes	Emerging sustainable technology
Cost efficiency	Low-cost reagents, high purification costs	Higher initial cost, fewer purification steps

**Table 3:** Comparative table of green-synthesized linoleic acid derivatives.

Derivative	Flavor Attributes	Nutritional Functions	Food Applications
Conjugated linoleic acid (CLA)	Rich, creamy, nutty; enhances umami and mouthfeel	Reduces body fat, modulates insulin, anti-inflammatory	Dairy products, functional beverages, snacks
13-hydroxy-octadecadienoic acid (13-HODE)	Grassy, green notes; precursor to lactones	PPARγ agonist, antioxidant, immunomodulatory	Fermented foods, emulsified sauces, spreads
9-hydroxy-octadecadienoic acid (9-HODE)	Mildly nutty; supports buttery flavors	Lipid metabolism regulation, anti-inflammatory	Bakery items, cereal bars, and plant-based milks
Epoxy-linoleic acid (EpOME)	Volatile, fruity notes can enhance aroma	Neuroprotective, vascular modulator	Energy drinks, neurosupportive supplements
13-oxo-octadecadienoic acid (13-KODE)	Earthy, roasted, savory; interacts in Maillard reactions	Anti-inflammatory, supports oxidative stress response	Savory sauces, roasted snacks, and clinical nutrition
